# The Influence of Genetic Stability on *Aspergillus fumigatus* Virulence and Azole Resistance

**DOI:** 10.1534/g3.117.300265

**Published:** 2017-11-17

**Authors:** Thaila Fernanda dos Reis, Lilian Pereira Silva, Patrícia Alves de Castro, Pollyne Borborema Almeida de Lima, Rafaela Andrade do Carmo, Marjorie Mendes Marini, José Franco da Silveira, Beatriz Henriques Ferreira, Fernando Rodrigues, Iran Malavazi, Gustavo H. Goldman

**Affiliations:** * Faculdade de Ciências Farmacêuticas de Ribeirão Preto, Universidade de São Paulo, Ribeirão Preto 14040-903, Brazil; † Departamento de Microbiologia, Imunologia e Parasitologia, Escola Paulista de Medicina, Universidade Federal de São Paulo, 04023-062 Brazil; ‡ Life and Health Sciences Research Institute (ICVS), School of Medicine, University of Minho, Braga, Portugal and ICVS/3B's - PT Government Associate Laboratory Braga/Guimarães, 4710-057 Portugal; § Departamento de Genética e Evolução, Centro de Ciências Biológicas e da Saúde, Universidade Federal de São Carlos, São Paulo 13565-905, Brazil

**Keywords:** *Aspergillus fumigatus*, ATM, ATR, azoles, DNA damage, *Galleria mellonela*, genetic instability, PFGE, virulence, voriconazole

## Abstract

Genetic stability is extremely important for the survival of every living organism, and a very complex set of genes has evolved to cope with DNA repair upon DNA damage. Here, we investigated the *Aspergillus fumigatus* AtmA (Ataxia-telangiectasia mutated, ATM) and AtrA kinases, and how they impact virulence and the evolution of azole resistance. We demonstrated that *A. fumigatus atmA* and *atrA* null mutants are haploid and have a discrete chromosomal polymorphism. The Δ*atmA* and Δ*atrA* strains are sensitive to several DNA-damaging agents, but surprisingly both strains were more resistant than the wild-type strain to paraquat, menadione, and hydrogen peroxide. The *atmA* and *atrA* genes showed synthetic lethality emphasizing the cooperation between both enzymes and their consequent redundancy. The lack of *atmA* and *atrA* does not cause any significant virulence reduction in *A. fumigatus* in a neutropenic murine model of invasive pulmonary aspergillosis and in the invertebrate alternative model *Galleria mellonela*. Wild-type, Δ*atmA*, and Δ*atrA* populations that were previously transferred 10 times in minimal medium (MM) in the absence of voriconazole have not shown any significant changes in drug resistance acquisition. In contrast, Δ*atmA* and Δ*atrA* populations that similarly evolved in the presence of a subinhibitory concentration of voriconazole showed an ∼5–10-fold increase when compared to the original minimal inhibitory concentration (MIC) values. There are discrete alterations in the voriconazole target Cyp51A/Erg11A or *cyp51*/*erg11* and/or Cdr1B efflux transporter overexpression that do not seem to be the main mechanisms to explain voriconazole resistance in these evolved populations. Taken together, these results suggest that genetic instability caused by Δ*atmA* and Δ*atrA* mutations can confer an adaptive advantage, mainly in the intensity of voriconazole resistance acquisition.

Genetic stability is extremely important for the survival of every living organism, and a very complex set of genes has evolved to cope with DNA repair upon DNA damage. These genes encode proteins that sense DNA damage, transduce the signal, and repair the DNA damage. In eukaryotes, the most important DNA damage signal transducers are the AtmA and Rad53-related (ATR) protein kinases, and two other downstream kinases: checkpoint kinases 1 and 2 (Chk1 and Chk2) (Awasthi *et al.* 2015; Yazinski and Zou 2016). Mammalian ATM and ATR are paralogous phosphatidyl-3-kinase (PI-3 kinase)-related protein kinases (PIKK) that lack lipid kinase activity, but phosphorylate substrate proteins on Ser or Thr residues that are followed by Gln (SQ or TQ motifs) (Shiloh 2001, 2003; Bakkenist and Kastan 2004; McKinnon 2004). These kinases possess both overlapping and distinct roles in the regulation of this response, and phosphorylate multiple targets that act collectively to maintain the genome integrity. Upon their activation, both ATM and ATR upregulate cell cycle checkpoint pathways, inducing cell cycle arrest and DNA repair. They respond to different kinds of DNA damage: ATM responds to DNA double-strand breaks (DSBs) (Paull 2015), while ATR is responsible for the integrity of replicating chromosomes (Branzei and Foiani 2008). ATR is also activated by DSBs, but through a mechanism that depends on ATM and the MRE11–RAD50–NBS1 (MRN) complex (Doksani *et al.* 2009; Jazayeri *et al.* 2006).

Plant and human fungal pathogens are continuously exposed to host defenses that affect their genetic stability, such as reactive oxygen and nitrogen species (Shen *et al.* 2017; Drummond *et al.* 2014). Moreover, opportunistic pathogens are also constantly exposed to toxins that can cause potential DNA damage, which are produced by competitor microorganisms. Fungi are normally haploid, but eventually different nuclei can fuse by sexual processes allowing the formation of diploid nuclei, while ploidy reduction can occur via meiosis (Ene and Bennett 2014). The different ploidy states can affect genome stability and gene dosage, influencing gene expression and the interaction with the host cells (Bennett *et al.* 2014). Mechanisms of genomic stability and DNA repair are essential for the maintenance of different fungal ploidy states. In fungi, ploidy can be changed not only in the sexual cycles by meiosis and fertilization, but also by other processes such as endoreduplication without subsequent segregation of chromosomes and in the parasexual cycle by ploidy reduction through an aneuploidization mechanism (Bennett *et al.* 2014; Ene and Bennett 2014; Bennett and Turgeon 2016).

Interestingly, these ploidy change mechanisms in fungi dramatically affect their phenotypes yielding, for instance, *Candida albicans* and *Cryptococcus neoformans* drug-resistant clinical isolates (Berman 2010; Pavelka *et al.* 2010; Semighini *et al.* 2011; Sheltzer and Amon 2011; Ni *et al.* 2013; Selmecki *et al.* 2006, 2008; Sionov *et al.* 2010, 2013; Ngamskulrungroj *et al.* 2012a,b). Genome sequencing of *C. neoformans* strains isolated from a relapsed patient before and after antifungal treatment revealed a chromosomal rearrangement and a base pair mutation in the *AVC1* gene, which is important for controlling many virulence traits (Chow *et al.* 2012). Not only chromosomal polymorphism can affect fungal drug resistance and virulence, but also mutations in genes essential for the maintenance of genome stability. Loss-of-heterozygosity events in diploid strains of the fungal pathogen *C. albicans* are also important for phenotypic diversity (Bennett *et al.* 2014). *C. glabrata* harboring mutations in the mismatch repair gene *MSH2* promotes the acquisition of resistance to multiple antifungals, as has been observed at high frequency in clinical isolates (Healey *et al.* 2016a,b). *Cr. neoformans MSH2*, *MLH1*, and *PMS1* (mismatch repair pathway) mutations increased fungal growth as detected in a lung assay of cryptococcosis (Liu *et al.* 2008). Recently, it was demonstrated that mismatch repair of DNA replication errors is important for *Cr. neoformans* microevolution into the host (Boyce *et al.* 2017).

In pathogenic filamentous fungi, there is very little information about the importance of genomic stability on population structure, drug resistance, and virulence. *Aspergillus fumigatus* is a very important opportunistic fungal pathogen that causes invasive aspergillosis, a disease that has a mortality rate of up to 90% in immunocompromised patients. Treatment with triazoles is the most important therapeutic strategy to control *A. fumigatus* infection; however, epidemiological research has indicated that the prevalence of azole-resistant *A. fumigatus* isolates has increased significantly over the last decade (Meis *et al.* 2016; Tashiro and Izumikawa 2016; Verweij *et al.* 2016; Garcia-Rubio *et al.* 2017). The most common resistance mechanisms are associated with substitutions in the target Cyp51A protein, tandem repeat sequence insertions at the *cyp51A* promoter, and overexpression of the ABC transporter Cdr1B (Hagiwara *et al.* 2016). More recently, environmental azole-resistant strains harboring the association of a tandem repeat sequence and punctual mutation of the Cyp51A gene (TR34/L98H and TR46/Y121F/T289A) have become widely disseminated all over the world (Hagiwara *et al.* 2016). Nonetheless, none of these studies have addressed the impact of genomic stability or DNA repair on *A. fumigatus* azole-resistance and virulence. Here, considering the importance of ATM and ATR in genomic stability and the activation of DNA repair mechanisms, we decided to investigate if ATM and ATR homologs (named AtmA and AtrA) are important for virulence and the evolution of drug resistance in *A. fumigatus*. Our results indicate that AtmA and AtrA collaborate for *A. fumigatus* genomic stability. However, *A. fumigatus* mutants (Δ*atmA* and Δ*atrA*) are able to keep their virulence, accompanied by the evolution of voriconazole resistance to higher levels than in the wild-type strain. Surprisingly, Cyp51A mutations or overexpression of *cyp51* and/or Cdr1B efflux transporter overexpression are not involved in most of the voriconazole-resistant strains, suggesting that additional mechanisms underlying the evolution of drug resistance prevail in these genetically unstable strains.

## Materials and Methods

### Ethics statement

The principles that guide our studies are based on the Declaration of Animal Rights ratified by the UNESCO in January 27, 1978 in its 8th and 14th articles. All protocols adopted in this study were approved by the local ethics committee for animal experiments from the University of São Paulo, Campus of Ribeirao Preto (Permit Number: 08.1.1277.53.6; Studies on the interaction of *A. fumigatus* with animals). Groups of five animals were housed in individually ventilated cages, and were cared for in strict accordance with the principles outlined by the Brazilian College of Animal Experimentation and the Guiding Principles for Research Involving Animals and Human Beings, American Physiological Society. All efforts were made to minimize suffering. Animals were clinically monitored at least twice daily and humanely killed if moribund (defined by lethargy, dyspnea, hypothermia, and weight loss). All stressed animals were killed by cervical dislocation.

### Strains, media, and growth conditions

The *A. fumigatus* parental strain used in this study was Af293 (*pyrG−*). A list of the strains used in this study is described in Supplemental Material, Table S1. All the constructed mutants were grown at 37° in either MM [1% glucose, 50 ml of a 20× salt solution (120 g/L NaNO_3_, 10.4 g/L KCl, 30 g/L KH_2_PO_4_, and 10.4 g/L MgSO_4_), and 1 ml/L of trace elements, pH 6.5] or complete medium [YG: 2% glucose, 0.5% yeast extract, and 1 ml/L of trace elements (22.0 g/L ZnSO_4_, 11 g/L boric acid, 5 g/L MnCl_2_, 5 g/L FeSO_4_, 1.6 g/L CoCl_2_, 1.6 g/L CuSO_4_, 1.1 g/L (NH_4_)_2_MoO_4_, and 50 g/L ethylenediaminetetraacetic acid)]. The media were made with or without 2% agar in order to obtain the solid or liquid medium, respectively. Additionally, uridine and uracil (1.2 g/L each) were added as a nutritional supplement when necessary, originating YUU (YG + UU) and MM + UU media, respectively. Expression of the atmA gene under the control of the *niiA* promoter was regulated by nitrogen source: promoter repression was achieved in modified minimal medium (AMM) (1% w/v glucose and 2% w/v agar) supplemented with ammonium tartrate, while induction was achieved in AMM plus sodium nitrate, according to the method of Punt *et al.* (1991).

### Identification of the A. fumigatus AtmA and AtrA, and construction of null mutants

The *A. fumigatus* AtmA and AtrA orthologs were identified through a BLASP search of the *A. fumigatus* database using the *A. nidulans* AtmA and AtrA sequences as queries. Single putative orthologs were identified for each gene: Afu5g12660 (AtmA) and Afu4g04760 (AtrA). AtmA and AtrA protein organization was analyzed by using the SMART interface (http://smart.embl-heidelberg.de/). DNA manipulations and standard genetic approaches were performed according to Sambrook and Russell (2001). The gene replacement cassette was constructed by *in vivo* recombination using *Saccharomyces cerevisiae* as a tool (Colot *et al.* 2006) and the target genes were replaced by the prototrophic marker gene *pyrG*. Briefly, ∼1.0 kb regions on either side of the *atmA* and *atrA* genes were PCR-amplified from the gDNA of the Af293 strain using specific primers: *atmA* (primers P1 and P2) and *atrA* (primers P5 and P6). Similarly, the 3′-UTR region of each gene was amplified using primers P3 and P4 (for *atmA*) and primers P7 and P8 (for *atrA*). The auxotrophic marker *pyrG* was amplified from pCDA21 plasmid (primers P9 and P10) (Table S2). The individual DNA fragments (5′- and 3′-UTRs plus *pyrG*) were cotransformed with the *Bam*HI–*Eco*RI-cut pRS426 plasmid into *S. cerevisiae* SC9721 strain using the lithium acetate method (Schiestl and Gietz 1989). The recombinant yeast candidates were selected in solid YNB-URA medium (7 g/L yeast nitrogen base without amino acids, 0.05 g/L histidine, 0.1 g/L lysine, 0.1 g/L leucine, 0.1 g/L tryptophan, and 2% agar). gDNA of the yeast candidates was extracted, and the deletion cassettes were PCR-amplified using specific primers (P1 and P4 for *atmA* and P5 and P8 for *atrA*, Table S2) and transformed into the *A. fumigatus* Af293 pyrG− background strain (Osmani *et al.* 1987). The PCR amplifications were performed using Phusion High-Fidelity DNA polymerase (New England Biolabs) or TaKaRa Ex Taq DNA Polymerase (Clontech). Positive *A. fumigatus* candidates were purified in a selective medium without uridine/uracil, and the gDNA was extracted (Goldman *et al.* 1995) and checked by southern blot using the AlkPhos Direct Labeling and Detection System (GE Healthcare Life Sciences), according to the manufacturer’s protocol. Two independent transformants for each gene deletion mutant were selected for further analysis. To construct the conditional double mutant Δ*atrA1 niiA*::*atmA*, the Δ*atrA1* strain was treated with 5-fluoroorotic acid, aiming to get a Δ*atrA1* pyrG− strain. Then, the Δ*atrA* pyrG− strain was transformed with the *niiA*::*atmA* cassette (pyrG+). Briefly, the 5′-UTR fragment and the atmA gene were PCR-amplified from Af293 gDNA using the primers P1/P11 and P12/P13, respectively. The *pyrG*::*niiA* fragment was PCR-amplified from the *niiA*::*ypkA* strain (Colabardini *et al.* 2013) using the specific primers *P18/P19*. The replacement cassette was also constructed in *S. cerevisiae* (exactly as described above) and the *A. fumigatus* transformation was processed by using Δ*atrA* pyrG− as a background strain. The conditional double mutant was selected in presence of AMM + 0.2 M NaNO_3_ and checked in the presence of AMM + 0.2 M (induces the promoter) or AMM + 0.2 or 0.4 M NH_4_ tartrate (represses the promoter). Once more, two independent transformants were selected for further analysis. gDNA extractions from *A. fumigatus* and *S. cerevisiae* were performed according to the methods of Goldman *et al.* (1995) and Schiestl and Gietz (1989), respectively.

### Measurements of DNA content per cell

Conidia were collected, centrifuged (and 13,000 rpm for 3 min), and washed with sterile 1× phosphate-buffered saline (PBS) (8 g NaCl, 0.2 g KCl, 1.44 g Na_2_HPO_4_, and 0.24 g KH_2_PO_4_ per liter of sterilized water). For cell staining, the protocol described by Almeida *et al.* (2007) was followed with modifications. Overnight fixation with 70% ethanol (v/v) at 4° was carried out. Following fixation, conidia were harvested, washed, and suspended in 850 µl of sodium citrate buffer (50 mM sodium citrate, pH = 7.5). Briefly, sonicated conidia were treated for 1 hr at 50° with RNase A (0.50 mg/ml; Invitrogen, Waltham, MA) and for 2 hr at 50° with proteinase K (1 mg/ml; Sigma-Aldrich, St. Louis, MO). Conidia were stained overnight with SYBR Green 10,000× (Invitrogen, Carlsbad, CA) diluted 10-fold in Tris-EDTA (pH 8.0), at a concentration of 2% (v/v) at 4°. Finally, Triton X-100 (Sigma-Aldrich) was added to samples at a final concentration of 0.25% (v/v). Stained conidia were analyzed in a Fluorescence-activated cell sorting (FACS) LSRII flow cytometer (Becton Dickinson, Franklin Lakes, NJ) with a 488 nm excitation laser. Signals from a minimum of 30,000 cells per sample were captured in the FITC channel (530 ± 30 nm) at a low flow rate of ∼1000 cells/sec, and an acquisition protocol was defined to measure forward scatter and side scatter on a four-decade logarithmic scale and green fluorescence (FITC) on a linear scale. FACS Diva was used as the acquisition software. Results were analyzed with FlowJo (Tree Star) software, version 10, and with Modfit LT Software (Verity Software House, Topsham, ME).

### Pulsed-field gel electrophoresis (PFGE), running conditions, and membrane transference

The agarose blocks containing chromosomal DNA from *A. fumigatus* isolates (wild strain and mutant strains) were submitted to. The Gene Navigator System (Pharmacia) was used under the conditions described by Sasaki *et al.* (2014) with minor modifications. The gels were prepared with Seaken agarose (FMC-Bioproducts), 1.1% in 1× TAE (Tris-acetate-EDTA) according to the electrophoretic run protocol, with a duration of 168 hr with constant voltage of 42 V at 10°. The best electrophoretic resolution was achieved with homogeneous pulses of 900 sec for 24 hr, 1800 sec for 24 hr, 2700 sec for 48 hr, 3600 sec for 48 hr, and 4500 sec for 24 hr, and were applied with interpolation. *Schizosaccharomyces pombe* and *S. cerevisiae* chromosomal DNA was used as the molecular size standard in each of the electrophoretic runs. After this process, the gel was incubated in ethidium bromide solution (0.5 µl/ml) for 30 min and photographed under ultraviolet (UV) light. The chromosomal bands were transferred to N+ nylon membranes (Amersham) using the VacuGene XL system (Pharmacia). Gels were deposited on the membrane and treated with three different solutions for 40 min each: solution 1 (depuration) containing 0.25 M HCl; solution 2 (denaturation) composed of 0.5 M NaOH and 1 M NaCl; and solution 3 (neutralization) containing Tris-base 1 M and 0.5 M NaCl pH 8.0. The gel was treated with 20× SSC (175.3 g/L NaCl and 88.2 g/L sodium citrate, pH 7.0) over 3 hr. The membranes were then irradiated with ultraviolet light (150 mJ) for DNA fixation using the GS Gene Linker UV Chamber (Bio-Rad) and stored at −20°.

### Phenotypic characterization of the ΔatmA and ΔatrA null mutants

A total of 10^5^ conidia of each strain tested were grown in solid MM in the presence or absence of camptothecin (CPT: 10, 25, and 50 µM), bleomycin (BLEO: 1, 2, and 4 µg/ml), methyl methanesulfonate (MMS: 0.001, 0.005, and 0.02%), 4- nitroquinoline-1-oxide (4-NQO: 0.01, 0.1, and 1 µg/ml), menadione (0.015 and 0.03 mM), and paraquat (0.25 mM). The plates were incubated for 120 hr at 37° and the radial growth was measured. The radial growth of each strain in the presence of drugs was normalized by the growth of each strain in MM without any drug. All plates were grown in triplicate and the average ± SD is plotted. Alternatively, 10^5^ conidia of each strain were inoculated in 500 µl of liquid MM medium supplemented or not with 1.5, 2, 2.5, 3, and 4 mM of H_2_O_2_.

### UV light viability assays

In order to determine the viability of the nondividing cells, a total of 100 fresh conidia for each strain were plated on the surface of solid YG using a spreader. Subsequently, the plates were irradiated with UV light using a UV Stratalinker 1800 (Stratagene, La Jolla, CA) and incubated at 37° for 48 hr to determine the UV sensitivity of G1 quiescent conidia (Bergen and Morris 1983). Additionally, to determine the UV survival of already dividing cells, 100 conidia for each strain were also spread on solid YG plates, but incubated for 4.5 hr at 37° before UV exposure (Malavazi *et al.* 2008). Cell viability was measured by determining the percentage of colonies on treated plates compared to the untreated controls. These experiments were done in triplicate for each strain and averages ± SD are shown.

### Mitosis assay

A total of 1 × 10^5^ conidia from wild-type, Δ*atmA*, and Δ*atrA* strains were inoculated in a 30 mm petri dish containing 3 ml of liquid YG supplemented with 100 mM of hydroxyurea (HU) (37°). After 5 hr of incubation, the germlings were released from cell cycle arrest by four washes with YG medium, and then incubated in fresh YG (without HU) at 37° for the indicated periods of time. For nuclei visualization, cells were treated for 5 min with 20 µg/ml of Hoechst 33342 (Invitrogen). After staining, coverslips were washed twice with PBS and analyzed on an Observer Z1 fluorescence microscope using a 100× objective oil immersion lens.

### RNA extraction and qPCR reactions

A total of 1 × 10^7^ conidia of the indicated *A. fumigatus* strains were inoculated in 50 ml of liquid MM and incubated in a reciprocal shaker at 37°, 180 rpm. After 16 hr of growth, the voriconazole was added to a final concentration of 0.35 µg/ml and incubated for an additional 4 hr. Then, mycelia were harvested by filtration, washed twice with sterile H_2_O, and immediately frozen in liquid nitrogen. For total RNA isolation, mycelia were ground in liquid nitrogen and the total RNA was extracted using Trizol (Invitrogen). The RNA integrity was analyzed using an Agilent 2100 Bioanalyzer system. For real-time PCR experiments, RNase-free DNase I treatment was carried out as previously described by Semighini *et al.* (2011) and cDNA was generated using the SuperScript III First Strand Synthesis system (Invitrogen) with oligo(dT) primers, according to the manufacturer’s instructions. All of the qPCR reactions were performed using an ABI 7500 Fast Real-Time PCR System (Applied Biosystems) and SYBR Green PCR Master Mix (Applied Biosystems). Primers P14 and P15 (Table S2) were used to evaluate the mRNA accumulation of the *cyp51A/erg11A* gene.

### Virulence analysis of the ΔatmA and ΔatrA mutants

In the murine model of pulmonary aspergillosis, lung histopathology, and fungal burden, outbreed female mice (BALB/c strain; body weight, 20–22 g) were housed in vented cages containing five animals. Mice were immunosuppressed and infected with 1 × 10^5^ conidia contained in 20 μl of suspension, according to a previously described protocol (Dinamarco *et al.* 2012). The statistical significance of the comparative survival values was calculated using Mantel–Cox and Gehan–Brestow–Wilcoxon log rank analysis, and the Prism statistical analysis package.

To investigate fungal burden and the histopathological pattern in the murine lungs after infection, mice were similarly immunosuppressed with cyclophosphamide but infected with 1 × 10^6^ conidia/20 μl of suspension. All the animals were killed 72 hr postinfection and the lungs were harvested. One of the lungs from each animal was immediately frozen in liquid nitrogen and used for fungal burden detection, while the other lung was immediately fixed in 10% formaldehyde solution and subjected to histopathology processing.

Larvae of *Galleria mellonella* were obtained by breeding adult moths (Fuchs *et al.* 2010) weighing 275–330 mg, kept under starvation in petri dishes at 37° in darkness for 24 hr prior to infection. All the selected larvae were in the final (sixth) instar larval stage of development. Fresh conidia from each *A. fumigatus* strain were harvested from YAG plates in PBS solution and filtered through a Miracloth (Calbiochem). For each different strain, the spores were counted using a hemocytometer and the stock suspension was done at 2 × 10^8^ conidia/ml. The viability of the administered inoculum was determined by plating a serial dilution of the conidia on YAG medium at 37°. A total of 5 μl (1 × 10^6^ conidia/larva) from each stock suspension was inoculated per larva. The control group was composed of larvae inoculated with 5 μl of PBS to observe the killing due to physical trauma. The inoculum was performed by using Hamilton syringe (7000.5KH) via the last left proleg. After infection, the larvae were maintained in petri dishes at 37° in the dark and were scored daily. Larvae were considered dead by presenting the absence of movement in response to touch.

### Fungal burden

For DNA extraction, lungs were ground in liquid nitrogen, homogenized by vortexing with glass beads for 10 min, and DNA was extracted via the phenol–chloroform method. DNA quantity and quality was assessed using a NanoDrop 2000 spectrophotometer (Thermo Scientific). At least 500 μg of total DNA from each sample was used for qPCR. A primer and a Lux probe (Invitrogen) was used to amplify the 18S rRNA region of *A. fumigatus* (primer, 5′-CTTAAATAGCCCGGTCCGCATT-3′; probe, 5′-CATCACAGACCTGTTATTGCCG-3′) and an intronic region of mouse glyceraldehyde-3-phosphate dehydrogenase (primer, 5′-CGAGGGACTTGGAGGACACAG-3′; probe, 5′-GGGCAAGGCTAAAGGTCAGCG-3′). Six-point standard curves were calculated using serial dilutions of gDNA from all the *A. fumigatus* strains used and the uninfected mouse lung. Fungal and mouse DNA quantities were obtained from the threshold cycle (*CT*) values from an appropriate standard curve. Fungal burden was determined as the ratio between picograms of fungal and micrograms of mouse DNA.

### Histopathology

After they had been fixed in 10% formaldehyde solution for the histopathology methodology, the lungs were diaphanized and embedded in paraffin, and then sliced in serial sections of ∼5 μm thickness. The staining was done with hematoxylin and eosin or GMS (Sigma-Aldrich GMS Kit). The slides were analyzed and the images were recorded using the microscope (Leica DM6000 B).

### Maintenance of genetic stability in atmA and atrA after sequential mitotic divisions

For the sequential mitotic divisions in solid MM without stress, the wild-type and null mutants Δ*atmA* and Δ*atrA* were divided into four independent populations, each originating a total of 20 different populations: WT0, WT1, WT2, WT3, Δ*Atm*1-0, Δ*Atm*1-1, Δ*Atm*1-2, Δ*Atm*1-3, Δ*Atm*2-0, Δ*Atm*2-1, Δ*Atm*2-2, Δ*Atm*2-3, Δ*Atr*1-0, Δ*Atr*1-1, Δ*Atr*1-2, Δ*Atr*1-3, Δ*Atr*2-0, Δ*Atr*2-1, Δ*Atr*2-2, and Δ*Atr*2-3. Then, a total of 1 × 10^8^ spores from each strain listed above (except the parentals: WT0, Δ*Atm*1-0, Δ*Atm*2-0, Δ*Atr*1-0, and Δ*Atr*2-0) were inoculated in solid MM and incubated for 72 hr at 37°. The spores were harvested in sterile water, washed twice, and plated again at a final concentration of 1 × 10^8^ in MM plates, then incubated for an additional 72 hr at 37°. The procedure above was repeated 10 times for each strain. By the end of the process, a total of 15 evolved populations (WT1, WT2, WT3, Δ*atm1*-1, Δ*atm*1-2, Δ*atm*1-3, Δ*atm*2-1, Δ*atm*2-2, Δ*atm*2-3, Δ*atr*1-1, Δ*atr*1-2, Δ*atr*1-3, Δ*atr*2-1, Δ*atr*2-2, and Δ*atr*2-3) were obtained and used for further assays.

For the sequential mitotic divisions in solid MM in the presence of voriconazole, the wild-type and null mutants Δ*atmA* and Δ*atrA* were divided into four independent populations, each originating a total of 20 different populations: WT0, WTV1, WTV2, WTV3, Δ*Atm*1-0, Δ*Atm*1*V*1, Δ*Atm*1*V*2, Δ*Atm*1*V*3, Δ*Atm*2-0, Δ*Atm*2*V*1, Δ*Atm*2*V*2, Δ*Atm*2*V*3, Δ*Atr*1-0, Δ*Atr*1*V*1, Δ*Atr*1*V*2, Δ*Atr*1*V*3, Δ*Atr*2-0, Δ*Atr*2*V*1, Δ*Atr*2*V*2, and Δ*Atr*2*V*3. Then, a total of 1 × 10^8^ spores from each strain listed above (except the parentals: WT0, Δ*Atm*1-0, Δ*Atm*2-0, Δ*Atr*1-0, and Δ*Atr*2-0) were inoculated in solid MM plus a subinhibitory concentration of voriconazole (0.2 µg/ml) and incubated for 5 d at 37°. The spores were harvested in sterile water, washed twice, and plated again at final concentration of 1 × 10^8^ in MM plates supplemented with voriconazole (0.2 µg/ml), and the incubated for additional 5 d at 37°. The procedure above was repeated five times for each strain, except the parentals (WT0, Δ*Atm*1-0, Δ*Atm*2-0, Δ*Atr*1-0, and Δ*Atr*2-0). By the end, a total of 15 evolved populations (WTV1, WTV2, WTV3, Δ*Atm*1*V*1, Δ*Atm*1*V*2, Δ*Atm*1*V*3, Δ*Atm*2*V*1, Δ*Atm*2*V*2, Δ*Atm*2*V*3, Δ*Atr*1*V*1, Δ*Atr*1*V*2, Δ*Atr*1*V*3, Δ*Atr*2*V*1, Δ*Atr*2*V*2, and Δ*Atr*2*V*3) were obtained and used for further assays.

### MIC

The susceptibility of the indicated strains was assessed by using antifungal MIC or minimal effective concentration (MEC) methods (Clinical and Laboratory Standards Institute 2008). The experiments were done in microtiter plates, which were filled up with 200 µl of liquid MM and inoculated with a total of 1 × 10^4^ conidia/well. Plates were incubated at 37° for 48 hr. MICs of the assayed drugs were determined visually as a no-growth endpoint at 48 hr of incubation. MECs gave the lowest caspofungin concentration that led to the growth of small, rounded, compact microcolonies compared the growth control (caspofungin-free MM) after 48 hr incubation. The different drugs were diluted with the following range: (i) caspofungin (0–2 µg/ml), (ii) voriconazole (0–2 µg/ml), and posaconazole (0–2 µg/ml). Three repetitions were performed for each treatment.

### Data availability

Strains are available upon request. The authors state that all data necessary for confirming the conclusions presented in the article are represented fully within the article.

## Results

### Molecular and functional characterization of A. fumigatus ATM and ATR orthologs

*A. nidulans* AtmA and AtrA orthologs were used as queries to identify the *A. fumigatus* AtmA and AtrA orthologs: Afu5g12660 (*e*-value: 00; identity: 65.7%; and similarity: 78.6%) and Afu4g04760 (*e*-value: 00; identity: 64.4%; and similarity: 78.3%), respectively. *A. fumigatus atmA* and *atrA* encode putative proteins with 2895 and 2451 amino acids and molecular weights of 325.7 and 276.1 kDa, respectively. The organization of protein domains analyzed by using the SMART interface (http://smart.embl-heidelberg.de/) showed that both *A. nidulans* and *A. fumigatus* structure and organization are very conserved (Figure S1). The AtmA has the following domains: a TAN domain (telomere-length maintenance and DNA damage repair; 3.1e−63; SM0001342) from amino acids 1–153, a FAT domain (present in the PIK-related kinases; *e*-value: 4.3–6; PF2259) from amino acids 1969–2884, a PI3Kc domain (phosphoinositide 3-kinase, catalytic domain; *e*-value: 2.09e−81; SM000146, from amino acids 2567–2884, and a FATC domain (possibly playing a role in redox-dependent structural and cellular stability; *e*-value: 1.68–9; SM001343). AtrA has the following domains: a UME domain (found in nucleolar proteins; *e*-value: 2.43e−51; SM000802) from amino acids 905–1011, a FAT domain (*e*-value: 2.9–44) from amino acids 1570–1900, a PI3Kc domain (*e*-value: 1.35–93) from amino acids 2143–2440, and a FATC domain (*e*-value: 7.14–13) from amino acids 2419–2451.

**Figure 1:**
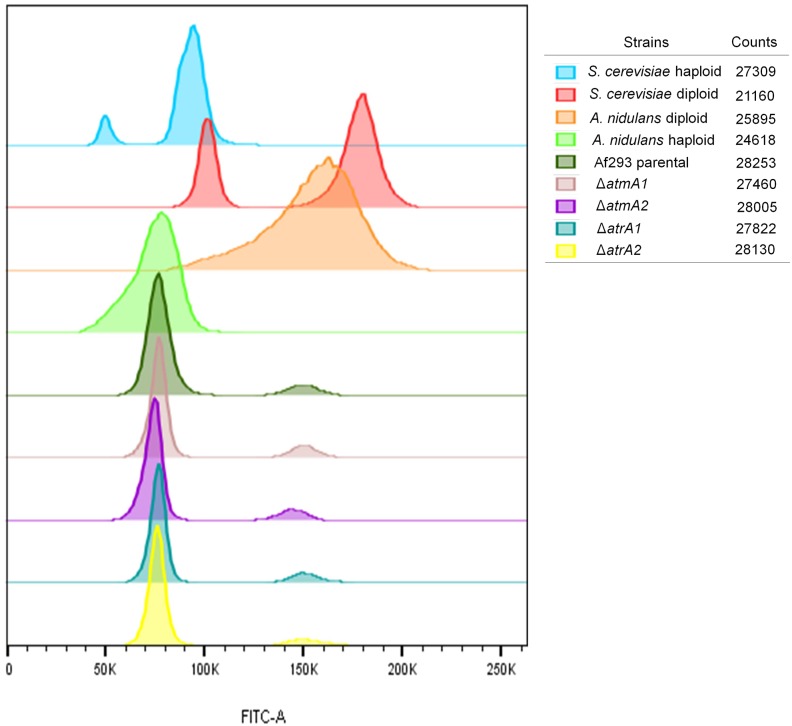
Fluorescence-activated cell sorting analysis of *A. fumigatus*, *A. nidulans* and *S. cerevisiae* DNA content. FITC, fluorescein isothiocyanate.

**Figure 2:**
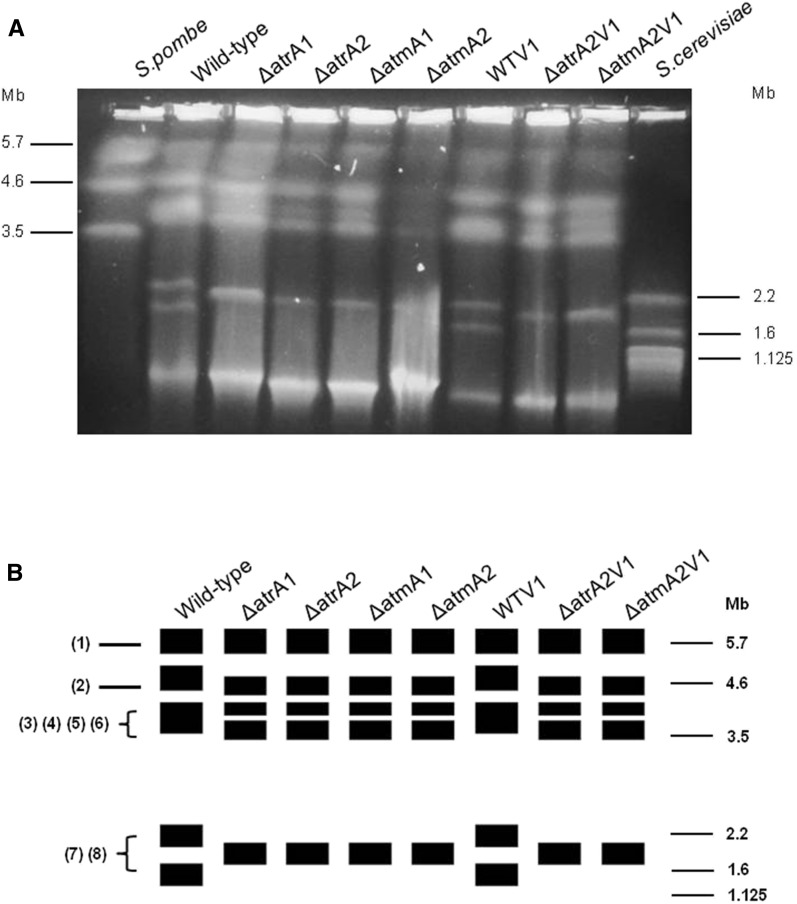
Karyotype polymorphism between wild-type strain and Δ*atmA* and Δ*atrA* mutants of *A. fumigatus*. (A) Chromosomal bands separated by pulsed-field gel electrophoresis and stained with ethidium bromide. *A. fumigatus* wild-type and mutants have five chromosomal bands. The smallest band (∼1.0 Mb) seen in the wild-type and mutants could correspond to a minichromosome. *Sc. pombe* and *S. cerevisiae* chromosomal bands were used as size standards indicated in megabases (Mb) at the right. *A. fumigatus* strains are: wild-type, mutants Δ*atm*A1-2, and Δ*atr*A1-2; and wild-type (WTV1) and mutants (Δ*atm*A2V1 and Δ*atr*A2V1) selected after grown with subinhibitory concentration of voriconazole. (B) Diagrammatic representation of karyotypes of wild-type mutants of *A. fumigatus*. The rectangles represent a unique distinguishable band visualized after staining with ethidium bromide. The thickness of the rectangles represents the proportional staining of each chromosomal band. At the left are indicated the *in silico* chromosomes of *A. fumigatus* assigned to the chromosomal bands.

In order to further investigate the role of AtmA and AtrA in *A. fumigatus*, Afu5g12660 and Afu4g04760 were targeted for entire gene deletion (Figure S2). Possibly due to the large size of the genes (plus the flanking regions), we were not able to complement the deletion strains with the corresponding wild-type genes. Thus, to discard the possibility of the occurrence of likely secondary mutations during the construction of deletion strains, we selected two independent transformants from each deletion experiment to pursue all our phenotypic analyses. First, we investigated whether *atmA* and *atrA* null mutations have caused any effect on ploidy and chromosome arrangement (Figure 1 and Figure 2). FACS was used to verify whether wild-type and mutant conidia have different ploidy (Figure 1). Haploid and diploid strains of *S. cerevisiae* and *A. nidulans* were used as controls of known cellular DNA content (Figure 1). The Af293 wild-type parental, Δ*atmA1* and -*A2*, and Δ*atrA1* and -*A2* mutant strains showed a DNA content consistent with a haploid distribution, suggestion that *atmA* and *atrA* null mutations have not affected the ploidy (Figure 1).

The haploid genome of *A. fumigatus* has 29,388,377 bp organized into eight chromosomes (www.aspgd.org). The molecular karyotype of *A. fumigatus* wild-type and *atmA* and *atrA* mutants was defined by PFGE. It comprises five chromosomal bands, whose sizes are slightly different between the wild-type and mutants (Figure 2). The wild-type karyotype has three chromosomal bands of ∼5.7, 4.9, and 3.9 Mb, and two smaller bands of 2.2 and 1.8 Mb (Figure 2). The mutants differ from the wild-type in the length of most of the chromosomal bands. They have four bands, the first ones with the same size (5.7 Mb) of the wild-type, and three others slightly shorter (4.6, 4.1, and 3.6 Mb) than the wild-type. The 4.1 Mb band could have arisen by a deletion event occurring in the 4.9 Mb band of the wild-type. In the mutants, the 2.2 Mb and 1.8 Mb bands have been replaced by a single band of 2.0 Mb, suggesting the occurrence of deletion and segmental duplication events in the 2.2 and 1.8 Mb bands, respectively. Finally, the shorter chromosomal band (∼1.0 Mb) seen in the wild-type and mutants could correspond to minichromosomes, which have been described in strains and progeny of crosses of several fungal species (Zolan 1995).

The fluorescence intensity varied between the bands, indicating that comigrating chromosomes are not necessarily homologous. Based on the chromosome length we tentatively assigned the *in silico* chromosomes (Chr) to the chromosomal bands of the wild-type: Chr1 (4.92 Mb) to the first band (5.7 Mb), Chr2 (4.84 Mb) to the second band (4.9 Mb), Chr3, Chr4, Chr5, and Chr6 (4.08, 3.92, 3.95 and 3.78 Mb) to the third band (3.9 Mb), Chr7 (2.06 Mb) to the fourth band (2.2 Mb), and Chr8 (1.83 Mb) to fifth band (1.8 Mb). Similarly, Chrs were assigned to the chromosomal bands of *atmA* and *atrA* mutants: Chr1 and Chr2 to the bands of 5.7 and 4.6 Mb, respectively; Chr3, Chr4, and Chr5 to the 4.1 Mb band; Chr6 to the 3.6 Mb band; and Chr7 and Chr8 to a single band of 2.0 Mb. In agreement with the FACS analysis, these results strongly suggest that chromosomal rearrangements found in null mutants did not affect the ploidy.

**Figure 3:**
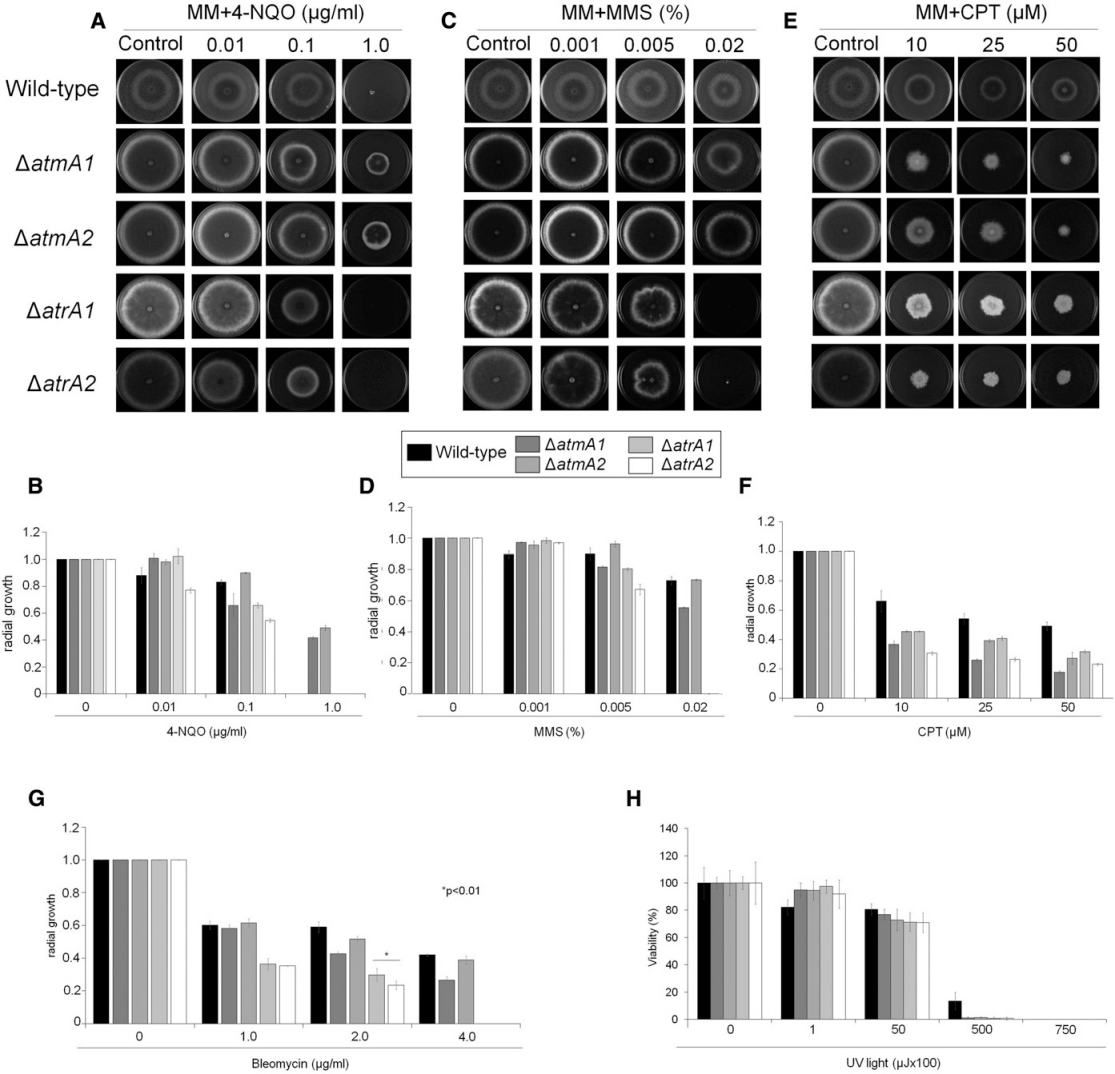
*A. fumigatus* Δ*atmA* and Δ*atrA* mutants have differential susceptibility to DNA-damaging agents. *A. fumigatus* wild-type, Δ*atmA1*, Δ*atmA2*, Δ*atrA1*, and Δ*atrA2* conidia (1 × 10^4^) were inoculated on MM plus different drug concentrations. (A and B) 4-NQO, (C and D) MMS, (E and F) CPT and (G) Bleomycin or (H) treated with UV light. Plates were incubated for 5 days at 37C. The results were expressed as the average of radial diameter of the treatment divided by the radial diameter of the control of three independent experiments ± SD (* *P* < 0.001, as determined by Student’s *t*-tests when comparing the treatment to the control). 4-NQO, 4-nitroquinoline oxide; CPT, camptothecin; MM, minimal media; MMS, methyl methanesulfonate; UV, ultraviolet.

We tested the influence of several DNA-damaging and oxidative stress agents on the growth of the wild-type and mutant strains (Figure 3 and Figure S3). The *atrA* and *atmA* mutants showed different resistance profiles against DNA-damaging agents. *In vitro* susceptibility testing showed that *atrA* mutants were 4-NQO and MMS susceptible, and that the growth was completely inhibited at concentrations of 1.0 µg/ml and 0.2% of 4-NQO and MMS, respectively (Figure 3). Interestingly, *atrA* mutants showed intermediate susceptibility to CPT. For *atmA* mutants, *in vitro* resistance against 4-NQO and MMS was found to be within the same range as that for the wild-type. The Δ*atmA* strains were about twofold more resistant than the wild-type to the quasi-mimetic UV light agent 4-NQO, while the Δ*atrA* strains (Figure 3A) were as sensitive as the wild-type (Figure 3, A and B). Both the Δ*atmA* and Δ*atrA* strains were more sensitive than the wild-type to CPT (Figure 3, E and F). The Δ*atrA* strains were more sensitive to BLEO than the wild-type strain (Figure 3G). Both the Δ*atmA* and Δ*atrA* strains were more sensitive to UV light (Figure 3H). Both Δ*atmA* and Δ*atrA* were more resistant than the wild-type strain to paraquat, menadione, and hydrogen peroxide (Figure S2, A–C).

**Figure 4:**
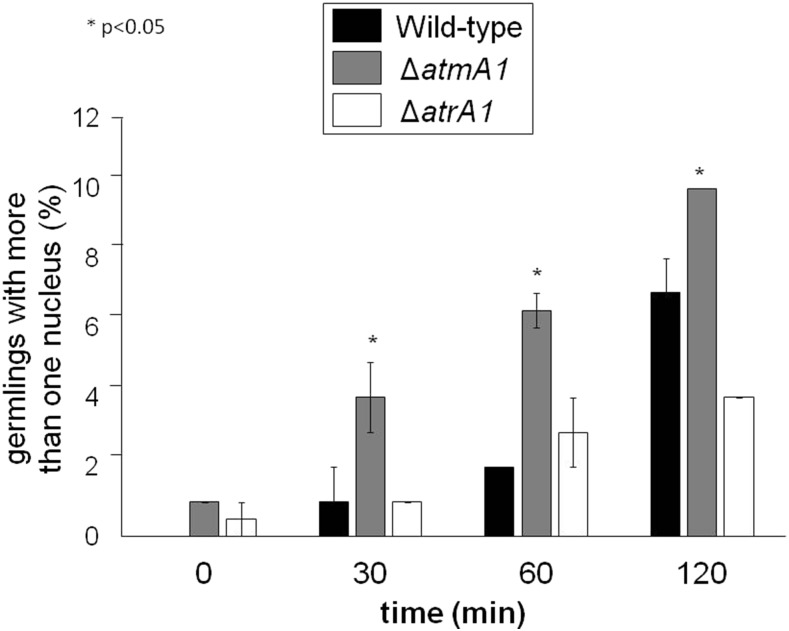
The Δ*atmA* mutant has a defective DNA replication checkpoint. Conidia were grown for 5 hr at 37° in complete medium (YG) + 100 mM HU then washed and incubated again in YG medium for different periods of time. The number of nuclei in each conidium was assessed by Hoescht staining (50 germlings were counted per each repetition, average of three independent experiments ± SD).

Upon DNA damage, the cell cycle is inhibited through the activation of cell cycle checkpoints. HU is an inhibitor of ribonucleotide diphosphate reductase, the rate-limiting enzyme in deoxyribonucleotide (dNTP) biosynthesis. The depletion of dNTPs activates the DNA replication checkpoint, slowing progression through S phase; in addition, the initiation of DNA replication in the presence of high levels of HU causes DSBs (Malavazi *et al.* 2008). Since HU is an effective inhibitor of DNA synthesis in *A. nidulans* (Bergen and Morris 1983), we investigated the ability of Δ*atmA* and Δ*atrA* to replicate DNA during a transient period of growth in the presence of HU. Previously, we have developed a mitosis assay (“DNA replication checkpoint response”; Fagundes *et al.* 2004) in which mitosis is monitored in the presence of HU for 5–7 hr. The number of nuclei is assessed by Hoescht staining, and if germlings have two or more nuclei after HU incubation, they are scored as defective in mitotic arrest (Figure 4). The Δ*atrA* strains had an intact replication checkpoint while the Δ*atmA* strains had defects in the intra-S-phase checkpoint (Figure 4).

**Figure 5:**
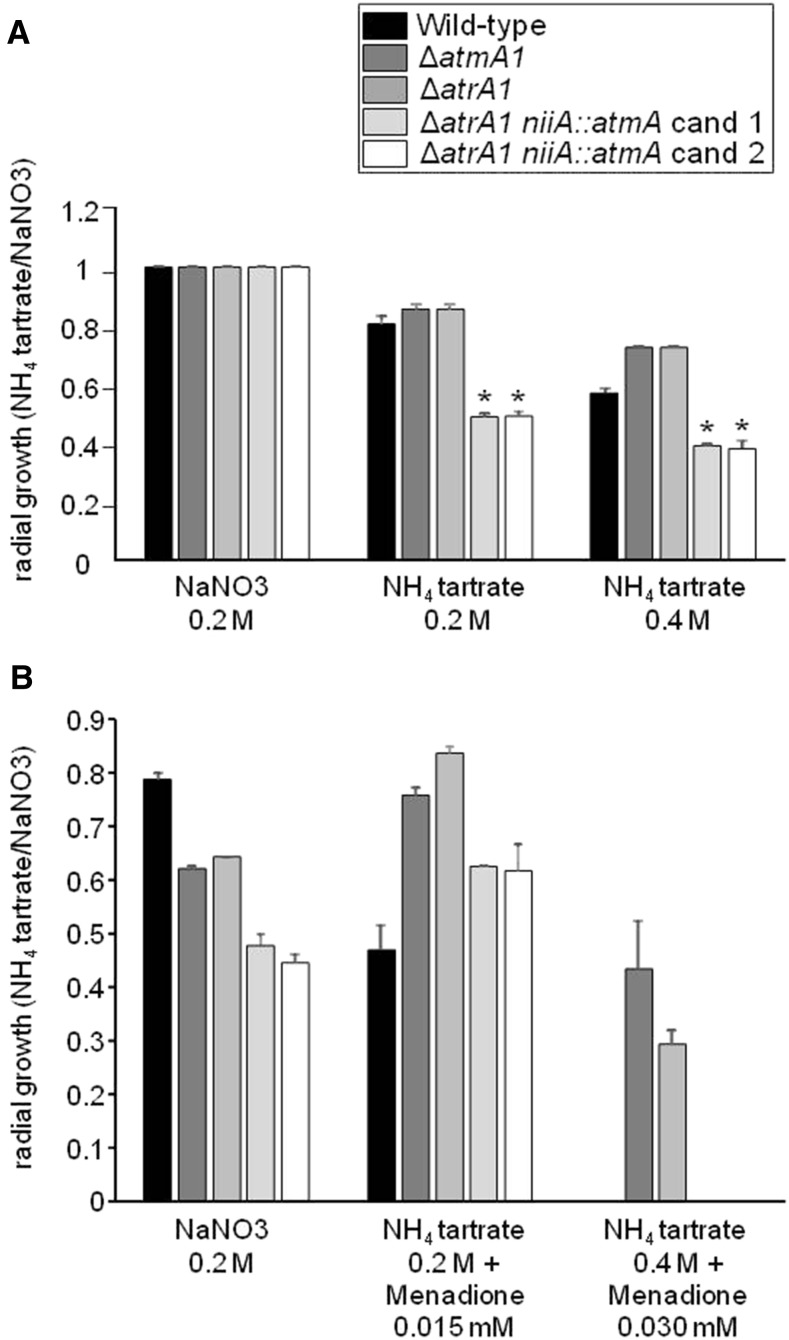
There is synthetic lethality between *A. fumigatus atmA* and *atrA*. *A. fumigatus* conidia (10^4^) were inoculated on minimal media (MM) + NaNO_3_ 0.2 M or MM + 0.2 or 0.4 M NH_4_ tartrate in the absence (A) and presence (B) of menadione 0.015 or 0.3 mM. Plates were incubated for 5 d at 37°. The results were expressed as the average of radial growth of MM + NH_4_ tartrate divided by MM + NaNO_3_ of three independent experiments ± SD (* *P* < 0.001, as determined by Student’s *t*-tests when compared the treatment to the control).

We have previously demonstrated that *A. nidulans* AtmA and UvsB^ATR^ show synthetic lethality (Malavazi *et al.* 2008). To verify whether there is a genetic interaction between *A. fumigatus* AtmA and AtrA, we further constructed two independent transformants for the conditional double mutant Δ*atrA1 niiA*::*atmA*. In this strain, *atmA* expression is under the control of the nitrite reductase genes promoter (*niia*), which is induced by sodium nitrate and repressed by ammonium tartrate (Punt *et al.* 1991). Indeed, *atmA* repression, along with Δ*atrA* deletion, led to an ∼50% reduction in *A. fumigatus* growth (Figure 5), indicating that a combination of AtmA and AtrA collaborate for fungal growth and that the double mutant may not be viable. Synthetic lethality was further confirmed by growth on the oxidative stressing agent menadione (Figure 5B). Both Δ*atmA* and Δ*atrA* are more resistant to menadione than the wild-type, while the two independent Δ*atrA1 niiA*::*atmA* candidates showed increased susceptibility to menadione (Figure 5B and Figure S3).

Taken together, these results suggest that AtmA and AtrA have redundant and separate functions during the DNA damage response in *A. fumigatus*. Furthermore, AtmA is important for the DNA replication checkpoint response, while the lack of one of these genes activates a strong response to agents that induce oxidative stress. We show that *atmA* is synthetic lethal with *atrA*, emphasizing the cooperation between both enzymes and their consequent redundancy.

### In vivo analysis of the influence of A. fumigatus ΔatmA and ΔatrA mutants on virulence

**Figure 6:**
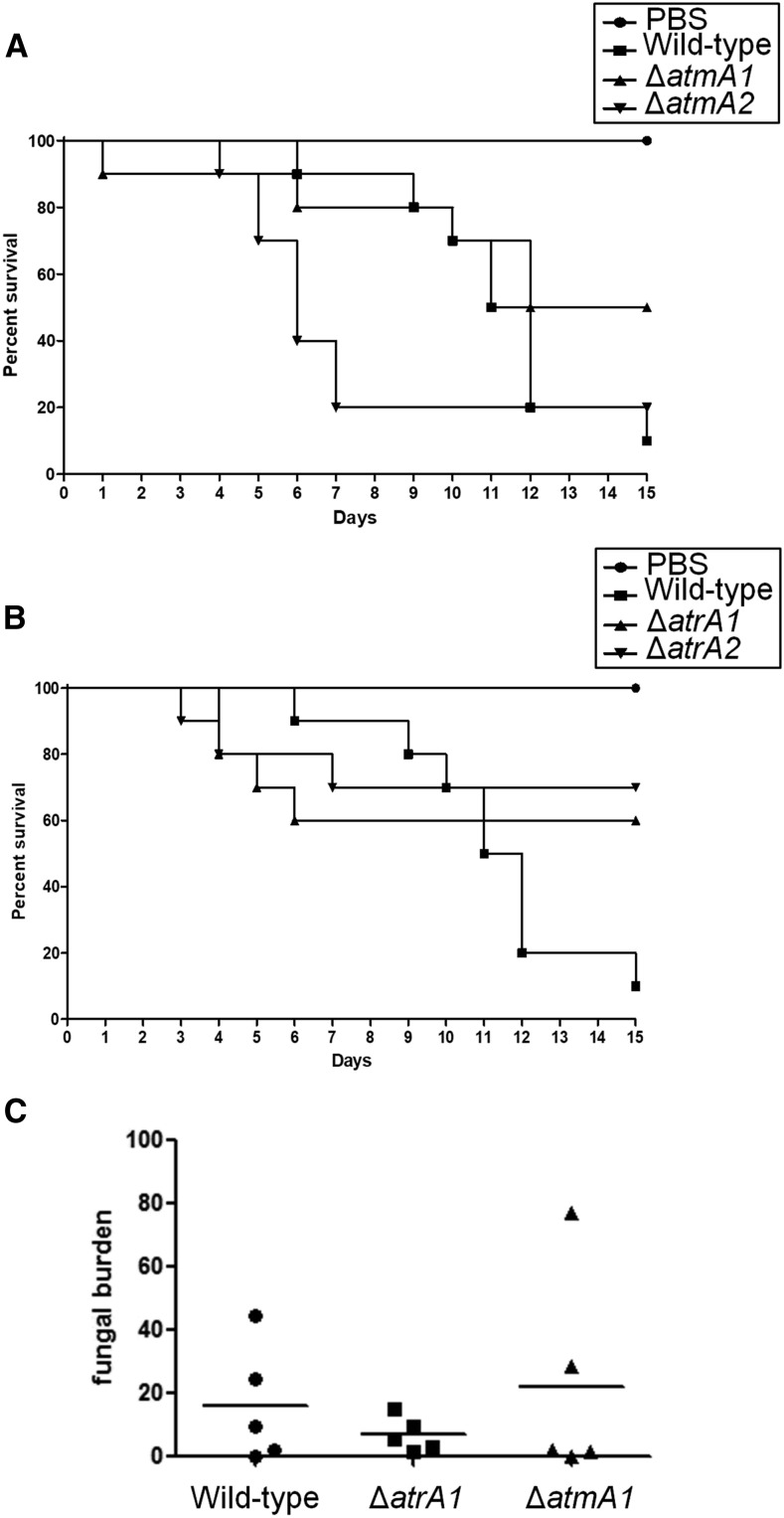
*A. fumigatus* Δ*atmA* and Δ*atrA* are still virulent. Comparative analysis of wild-type and mutant strains in a neutropenic murine model of pulmonary aspergillosis. Mice in groups of 10 per strain were infected intranasally with a 20 μl suspension of conidia at a dose of 10^5^ conidia. (A) Percent survival of Δ*atmA1* and Δ*atmA2* mutants compared to the wild-type strain. (B) Percent survival of Δ*atrA1* and Δ*atrA2* mutants compared to the wild-type strain. (C) Fungal burden quantification, determined by quantitative polymerase chain reaction from lungs obtained 72 hr postinfection. Results are expressed based on quantification of the 18S rRNA gene of *A. fumigatus* divided by the quantification of an intronic region of the mouse GAPDH gene. PBS, phosphate-buffered saline.

**Figure 7:**
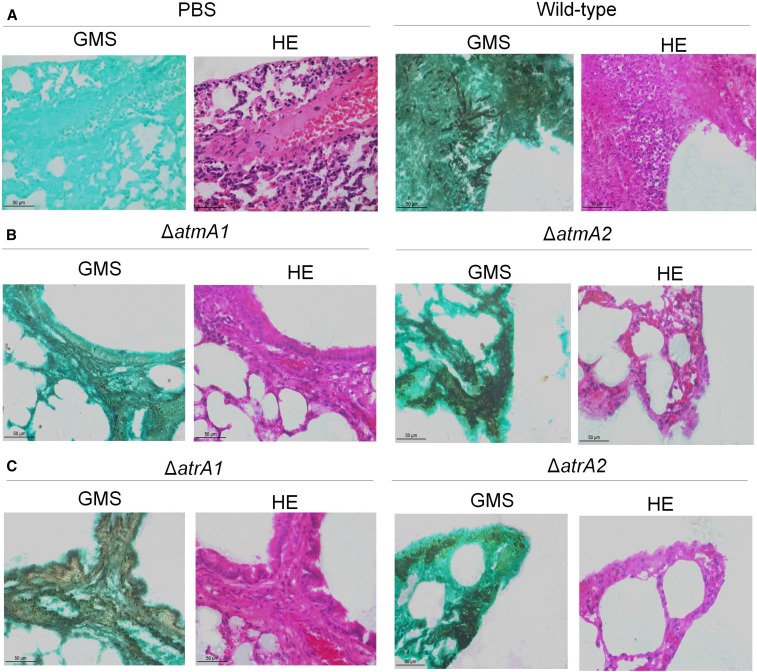
Histopathology of mice infected with *A. fumigatus* wild-type, and Δ*atmA* and Δ*atrA* mutant strains. (A) PBS and wild-type strain. (B) Δ*atmA1* and Δ*atmA2* mutants. (C) Δ*atrA1* and Δ*atrA2* mutants. PBS was used in the mock-infected animals. GMS and HE staining of lung sections of representative of infections. Bars, 50 µm. GMS, Grocott’s Methenamine Silver; HE, hematoxylin and eosin; PBS, phosphate-buffered saline.

In the neutropenic murine model of invasive pulmonary aspergillosis, infection by the wild-type and mutants (Δ*atmA1*, Δ*atmA2*, Δ*atrA1*, and Δ*atrA2*) resulted in 50–80% mortality 15 d postinfection, respectively (Figure 6, A and B). Although Δ*atrA1* and Δ*atrA2* virulence seem to be attenuated when compared to the wild-type, all isolates did not show a statistically significant difference according to the Mantel–Cox and Gehan–Brestow–Wilcoxon tests (Figure 6, A and B). Fungal burden confirmed these results (Figure 6C). Histopathological examination revealed that, at 72 hr postinfection, the lungs of mice infiltrated with PBS showed no signal of inflammation or pathogenesis, while mice infected with the wild-type strain contained multiple foci of invasive hyphal growth, which penetrated the pulmonary epithelium in major airways, while pockets of branched invading hypha originated from the alveoli (Figure 7A). Comparable phenotypes were observed for Δ*atmA1* and Δ*atmA2* strains (Figure 7B), and Δ*atrA1* and Δ*atrA2* strains (Figure 7C). These data strongly indicate that, when compared to the wild-type strain, the lack of *atmA* and *atrA* did not cause any significant virulence reduction in *A. fumigatus*.

In another set of experiments, we use the moth *G. mellonella* as an alternative animal model to compare the virulence between the mutants and wild-type. We verified if sequential mitotic divisions of the wild-type and Δ*atmA* and Δ*atrA* mutant strains without selective pressure could have an impact on virulence. First, we established three independent populations for each strain (WTO, WT1, WT2, WT3, ΔATM1-0, ΔATM1-1, ΔATM1-2, ΔATM1-3, ΔATM2-1, ΔATM2-2, ΔATM2-3, ΔATR1-0, ΔATR1-1, ΔATR1-2, ΔATR1-3, ΔATR2-0, ΔATR2-1, ΔATR2-2, and ΔATR2-3). Then, we transferred these populations through 10 conidia passages from each strain, except the parental ones (WT0, ΔATM1-0, ΔATM2-0, ΔATR1-0, and ΔATR2-0), on MM plates and grew them at 37° without any selective pressure (see Figure 9A, left panel). These evolved strains showed the same phenotypes related to growth, conidiation, and sensitivity to DNA damage agents as the original parental strains (data not shown).

**Figure 8:**
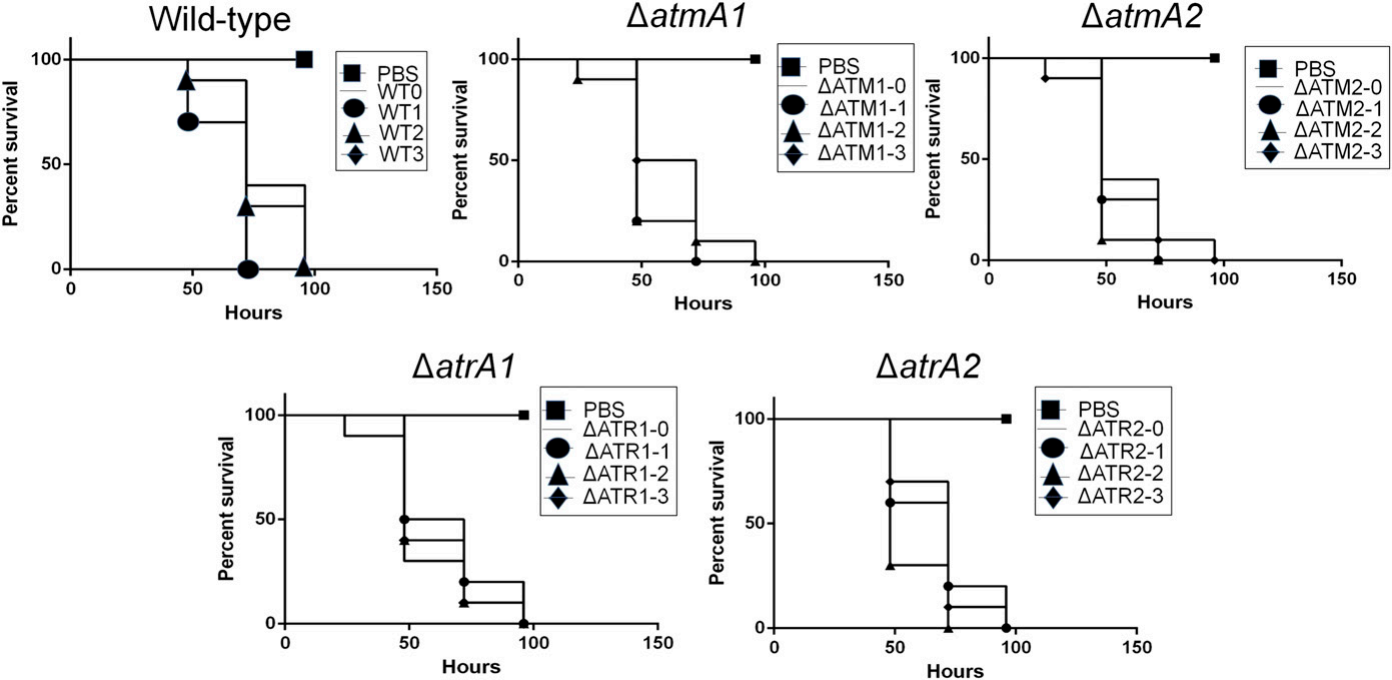
The Δ*atmA* and Δ*atrA* mutants are still virulent after several rounds of mitotic divisions. Comparative analysis of WT0, to -1 to -3; ΔATM1-0, to -1 to -3; ΔATM2-0, to -1 to -3; ΔATR1-0, to -1 to -3; and ΔATR2-0, to -1 to -3 strains in a *G. mellonella* animal model. Larvae in groups of 10 per strain were infected with h5 µl suspension of conidia at a dose of 1 × 10^6^/larva. PBS, phosphate-buffered saline.

Conidia of the original populations and the last-transferred populations had their virulence compared by inoculating them in *G. mellonella* larvae. In the *G. mellonella* model, infection of all the strains resulted in 100% mortality 72–100 hr postinfection (Figure 8). There was no statistical difference between all the strains (Mantel–Cox and Gehan–Brestow–Wilcoxon, *P* values > 0.05). These results strongly indicate that, in the presence of genetic instability caused by the absence of AtmA and AtrA, *A. fumigatus* can retain its virulence attributes in the *G. mellonella* model of infection.

### The influence of A. fumigatus ΔatmA and ΔatrA mutants on azole resistance

To investigate if the absence of AtmA and AtrA could impact azole resistance, we determined that the MIC for voriconazole and posoconazole in the *A. fumigatus* wild-type strain is 0.6 µg/ml. Interestingly, the Δ*atmA1* and -*A2* and Δ*atrA1* and -*A2* mutants have MICs of 0.4 and 0.6 µg/ml for voriconazole and posoconazole, respectively. The MEC for caspofungin for the wild-type and mutant strains is 0.125 µg/ml. We sequenced the *cyp51A/erg11A* (Afu4g06890) gene that encodes 14-α sterol demethylase, which is commonly mutated in drug resistant isolates [for a review, see Hagiwara *et al.* (2016)], in the wild-type and mutant strains (Table 1). We did not observe any amino acid substitutions in Erg11A in the wild-type strain when compared to the sequence that is described in the *Aspergillus* genome database (www.aspgd.org). In contrast, the Δ*atmA1* and -*A2* and Δ*atrA2* mutants have amino acid substitutions at V172M, T248N, E255D, K323Q, and K427E (Table 1). These results suggest that the deletion of the AtmA and AtrA genes is able to induce single nucleotide point mutations in the *erg11A* gene. However, it remains to be determined if the reduced MIC for voriconazole is due to the presence of these mutations.

**Table 1:** The *erg11A* sequence modifications observed in selected *A. fumigatus* ΔATM1, ΔATM2, ΔATR1, and ΔATR2 voriconazole-evolved populations

	Nucleotide Exchange with Amino Acid Change							Nucleotide Exchange Without Amino Acid Change		
Wild-type	Amino acid position	Y^46^	V^172^	T^248^	E^255^	K^323^	K^427^	G^89^	L^358^	C^454^
	Nucleotide sequence	_136_TAT_138_	_514_GTG_516_	_742_ACT_744_	_763_GAG_765_	_967_AAG_969_	_1279_AAG_1281_	_265_GAA_267_	_1072_TTG_1074_	_1360_TGC_1362_
WTV1	Amino acid position	ND	ND	ND	ND	ND	ND	ND	ND	ND
	Nucleotide sequence	ND	ND	ND	ND	ND	ND	ND	ND	ND
ΔATMA1V0	Amino acid position	ND	V^172^M	T^248^N	E^255^D	K^323^Q	K^427^E	G^89^	L^358^	C^454^
	Nucleotide sequence	ND	_514_ATG_516_	_742_AAT_744_	_763_GAC_765_	_967_CAG_969_	_1279_GAG_1281_	_265_GGG_267_	_1072_TTA_1074_	_1360_TGT_1362_
ΔATMA1VA	Amino acid position	Y^46^F	V^172^M	T^248^N	E^255^D	K^323^Q	K^427^E	G^89^	L^358^	C^454^
	Nucleotide sequence	_136_TTT_138_	_514_ATG_516_	_742_AAT_744_	_763_GAC_765_	_967_CAG_969_	_1279_GAG_1281_	_265_GGG_267_	_1072_TTA_1074_	_1360_TGT_1362_
ΔATMA1VB	Amino acid position	Y^46^F	V^172^M	T^248^N	E^255^D	K^323^Q	K^427^E	G^89^	L^358^	C^454^
	Nucleotide sequence	_136_TTT_138_	_514_ATG_516_	_742_AAT_744_	_763_GAC_765_	_967_CAG_969_	_1279_GAG_1281_	_265_GGG_267_	_1072_TTA_1074_	_1360_TGT_1362_
ΔATMA2V0	Amino acid position	Y^46^F	V^172^M	T^248^N	E^255^D	K^323^Q	K^427^E	G^89^	L^358^	C^454^
	Nucleotide sequence	_136_TTT_138_	_514_ATG_516_	_742_AAT_744_	_763_GAC_765_	_967_CAG_969_	_1279_GAG_1281_	_265_GGG_267_	_1072_TTA_1074_	_1360_TGT_1362_
ΔATMA2VC	Amino acid position	Y^46^F	V^172^M	T^248^N	E^255^D	K^323^Q	K^427^E	G^89^	L^358^	C^454^
	Nucleotide sequence	_136_TTT_138_	_514_ATG_516_	_742_AAT_744_	_763_GAC_765_	_967_CAG_969_	_1279_GAG_1281_	_265_GGG_267_	_1072_TTA_1074_	_1360_TGT_1362_
ΔATRA2V0	Amino acid position	Y^46^F	V^172^M	T^248^N	E^255^D	K^323^Q	K^427^E	G^89^	L^358^	C^454^
	Nucleotide sequence	_136_TTT_138_	_514_ATG_516_	_742_AAT_744_	_763_GAC_765_	_967_CAG_969_	_1279_GAG_1281_	_265_GGG_267_	_1072_TTA_1074_	_1360_TGT_1362_
ΔATRA2VA	Amino acid position	Y^46^F	V^172^M	T^248^N	E^255^D	K^323^Q	K^427^E	G^89^	L^358^	C^454^
	Nucleotide sequence	_136_TTT_138_	_514_ATG_516_	_742_AAT_744_	_763_GAC_765_	_967_CAG_969_	_1279_GAG_1281_	_265_GGG_267_	_1072_TTA_1074_	_1360_TGT_1362_
ΔATRA2VB	Amino acid position	Y^46^F	V^172^M	T^248^N	E^255^D	K^323^Q	K^427^E	G^89^	L^358^	C^454^
	Nucleotide sequence	_136_TTT_138_	_514_ATG_516_	_742_AAT_744_	_763_GAC_765_	_967_CAG_969_	_1279_GAG_1281_	_265_GGG_267_	_1072_TTA_1074_	_1360_TGT_1362_
ΔATRA2VC	Amino acid position	Y^46^F	ND	T^248^N	E^255^D	K^323^Q	K^427^E	G^89^	L^358^	C^454^
	Nucleotide sequence	_136_TTT_138_	ND	_742_AAT_744_	_763_GAC_765_	_967_CAG_969_	_1279_GAG_1281_	_265_GGG_267_	_1072_TTA_1074_	_1360_TGT_1362_

ND, not different from the *erg11A* wild-type strain.

**Figure 9:**
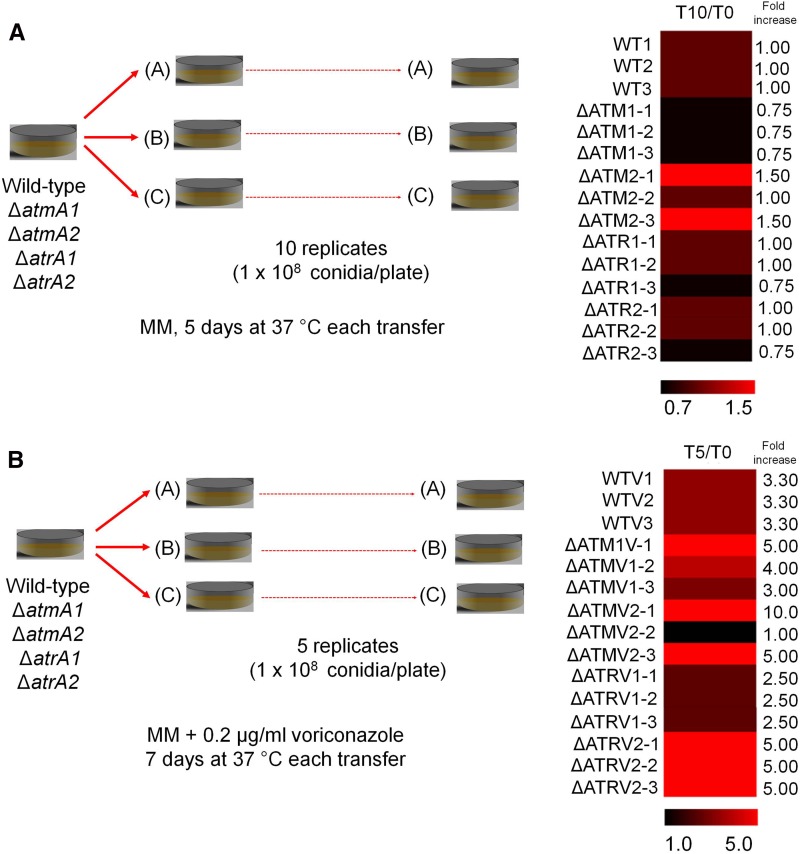
The *A. fumigatus* AtmA and AtrA mutants are important for the development of high resistance to voriconazole. (A) Left panel, conidia (1 × 10^8^/plate) of WT0, -1 to -3; ΔAtmA1-0, -1 to -3; ΔAtmA2-0, -1 to -3; ΔAtrA10-1, -1 to -3; and ΔAtrA20-2, -1 to -3 strains were transferred 10 times every 5 d to MM plates and incubated at 37°. Minimal inhibitory concentrations (MICs) were determined according to the Clinical and Laboratory Standards Institute (B) (CLSI, M38A2; http://clsi.org/) Left panel: conidia (1 × 10^8^/plate) of WTV0, -1 to -3; ΔATMA1V-0, to -A and -C; ΔATMA2V-0, to -A and -C; ΔATRA1V-0, to -A and -C; and ΔATRA2V-0, to -A and -C strains were transferred five times every 7 d to MM + 0.2 µg of voriconazole plates and incubated at 37°. (A and B) Right panels: MICs values of the strains after 10 and 5 transfers were divided by the parental strains (T10/T0 or T5/T0). Heat map shows the change in MIC values for the evolved strains in the absence or presence of voriconazole.

Subsequently, we measured the MICs to voriconazole in the populations that were previously transferred 10 times in MM in the absence of voriconazole (Figure 9A). We did not observe significant changes in drug resistance acquisition for the last set of transferred wild-type populations; however, the last set of Δ*atmA* and Δ*atrA* populations gained and lost 25–50% drug resistance, suggesting that the genetic instability caused by the mutations in the absence of selection does not dramatically affect voriconazole resistance status.

We also evolved three independent populations of the wild-type, Δ*atmA1*, Δ*atmA2*, Δ*atrA1*, and Δ*atrA2* strains on MM plates with a subinhibitory concentration of 0.2 µg/ml of voriconazole. Conidia of these three independent populations for each strain (WTVO, WTV1, WTV2, WTV3, ΔATMA1V0, ΔATMA1V1, ΔATMA1V2, ΔATMA1V3, ΔATMA2V0, ΔATMA2V1, ΔATMA2V2, ΔATMA2V3, ΔATRA1V0, ΔATRA1V1, ΔATRA1V2, ΔATRA1V3, ΔATRA2V0, ΔATRA2V1, ΔATRA2V2, and ΔATRA2V3) were transferred through five passages, except for the parental ones (WTVO, ΔATMAV0, and ΔATRAV0), on MM plates plus 0.2 µg/ml of voriconazole and grown at 37° (Figure 9B, left panel). After five passages, the three populations of the wild-type strain evolved 3.3-fold greater resistance to voriconazole, while 50% of the Δ*atmA* and Δ*atrA* populations evolved much greater voriconazole resistance (∼5–10-fold resistance, Figure 3B, right panel). The other 50% of the Δ*atmA* and Δ*atrA* populations evolved voriconazole resistance that was comparable (2.5-fold) or lower than the wild-type populations (for instance, see population ΔATMV2-2, Figure 9B). We also evaluated the occurrence of possible amino acid substitutions of Erg11A in selected mutants (Table 1). In comparison to the corresponding parental strains, the ΔATMA1VA, ΔatmA1VB, ΔATMA2VC, ΔATRA2VA, ΔATRA2VB, and ΔATRA2VC mutants showed a single Y^46^F amino acid substitution in Erg11A (Table 1). There is increased *erg11A* mRNA accumulation (∼five- to eightfold) when the WTV0 and selected mutant strains were grown in the presence of 0.35 µg/ml voriconazole (Figure S4A). In the presence of voriconazole, the *erg11A* mRNA accumulation is reduced ∼30% in the WTV1 and WTV2 mutants when compared to the wild-type strain (Figure S4A). In the absence of voriconazole, the *erg11A* mRNA accumulation is increased about threefold in ΔATM2V1 when compared to the corresponding parental strain (Figure S4B). Moreover, in the presence of voriconazole, ΔATM1V1, ΔATM1V2, and ΔATM2V1 strains have ∼20–30% more *erg11A* mRNA accumulation than the corresponding parental strain; surprisingly, ΔATM2V1 that has increased mRNA accumulation in the absence of voriconazole showed about threefold less expression than the corresponding parental strain (Figure S4B). In the presence of voriconazole, the ΔATR2V1, ΔATR2V2, and ΔATR2V3 strains showed ∼20% less *erg11A* mRNA accumulation than the corresponding parental strain and, interestingly, ΔATR2V0 showed ∼25% more *erg11A* mRNA accumulation than ΔATR1V0 (Figure S4C). We also investigated if the ABC transporter Cdr1B (Hagiwara *et al.* 2016) was overexpressed in these strains by using qRT-PCR (Figure S5). As expected, most of the wild-type and mutant strains had increased *cdr1B* mRNA accumulation in the presence of voriconazole (Figure S5, A–C). However, some of the evolved strains showed decreased *cdr1B* and discretely increased mRNA accumulation (Figure S5, A–C). These evolved strains have shown the same phenotypes regarding growth, conidiation, and sensitivity to DNA damage agents as the original parental strains (data not shown). The WTV1, ΔATR2V1, and ΔATMA2V1 strains showed the same chromosomal patterns and ploidy of their parental strains (Figure 1 and Figure S6).

Taken together, these results suggest that genetic instability caused by Δ*atmA* and Δ*atrA* mutations can confer an adaptive advantage, mainly in the intensity of voriconazole resistance acquisition. There are discrete alterations in the voriconazole target Cyp51A/Erg11A, or *cyp51A/erg11* and *cdr1B*, overexpression that do not seem to be the main mechanism to explain voriconazole resistance in these evolved populations.

## Discussion

Here, we have investigated whether two genetic determinants essential for genomic stability in eukaryotes, ATM and ATR, are important for *A. fumigatus* azole resistance and virulence. We have previously shown that *A. nidulans* AtmA^ATM^ loss-of-function caused synthetic lethality when combined with a mutation in UvsB^ATR^, suggesting that ATM and UvsB^ATR^ are genetically interacting and are probably partially redundant in terms of DNA damage sensing and/or repairing (Malavazi *et al.* 2008). The Δ*atmA* and Δ*atrA* mutants displayed differential sensitivity to different DNA-damaging agents; however, Δ*atrA* was more sensitive to BLEO, a DNA DSB-inducing agent. *A. nidulans* Δ*atmA* is more sensitive to DNA DSBs than Δ*atrA* (Malavazi *et al.* 2006, 2008). Interestingly, another important difference between *A. nidulans* and *A. fumigatus* is that both *A. fumigatus* Δ*atmA* and Δ*atrA* mutants were more resistant to oxidative stressing agents than the wild-type, suggesting that AtmA and AtrA modulate the oxidative stressing response in this species. Here, we also observed that *A. fumigatus* AtmA and AtrA genetically interact because the conditional repression of *atmA* in the Δ*atrA* background showed synthetic lethality. *Neurospora crassa atmA^mus-21^* and *atrA^mus-9^* are involved in the DNA damage response, normal growth, maintenance of chromosome integrity, and the regulation of different pathways; double conditional mutants for these two genes indicate that at least one of the pathways must be functional for survival (Wakabayashi *et al.* 2008, 2010). These results suggest that AtmA and AtrA have different, separate, and redundant functions in different fungal systems.

We have observed that Δ*atmA* and Δ*atrA* mutants are haploid and that there is limited chromosomal polymorphism in these mutants. We have not investigated the possible original events that were responsible for this chromosomal polymorphism; however, a striking observation is that these mechanisms are conserved in both Δ*atmA* and Δ*atrA*, since the observed chromosomal polymorphism is identical for all mutants, including those evolved under selective voriconazole pressure. Chromosome size differences between mutants ΔAtmA and ΔAtrA and the parental strain were relatively small (< 0.7 Mb), suggesting that chromosome rearrangements might be the result of DNA amplification/deletion events rather than large interchromosomal exchanges. These events could occur at the chromosome termini in which telomeric and subtelomeric regions are hotspots for recombination events in several unicellular microorganisms. The occurrence of chromosomal breaks in the chromosome ends could be followed by telomerase-mediated healing, which may generate new short telomeres. It is possible that these dramatic rearrangements reflect a role for AtmA and AtrA in telomeric and subtelomeric maintenance. In mammalian cells, ATM and ATR play an important role in telomere shortening and stabilization (Awasthi *et al.* 2015; Yazinski and Zou 2016). The *C. albicans mec1*Δ/Δ (Mec1p is the homolog of AtrA) mutant was also shown to exhibit an increase in genome instability in an assay for chromosome 1 integrity (Legrand *et al.* 2011).

*A. fumigatus* Δ*atmA* and Δ*atrA* are virulent in both a neutropenic murine model of invasive pulmonary aspergillosis and in *G. mellonela*. We performed sequential growth transfers of the wild-type and mutants, aiming to increase their mitotic instability and verifying if this could affect their virulence. However, we did not observed any virulence decrease in these derived mutant populations. Nevertheless, *C. albicans* mutants that affect homologous recombination or nonhomologous end-joining (NHEJ) repair of DNA DSBs, such as *LIG4* (which is the structural and functional homolog of both yeast and human ligase IV, involved in NHEJ) and *RAD52*, resulted in the attenuation of virulence in a murine model of candidiasis (Andaluz *et al.* 2001; Chauhan *et al.* 2005). These results emphasize once more that *A. fumigatus* AtmA and AtrA play redundant roles in DNA stability, growth, and the maintenance of virulence.

We also evaluated the influence of genetic stability on the evolution of voriconazole drug resistance. Upon selective pressure on voriconazole, selected Δ*atmA* and Δ*atrA* evolved strains have increased voriconazole resistance (∼4–10-fold more resistant when compared to the wild-type strain). In some selected mutants, we have identified a novel mutation in the Erg11A (Y^46^F), and/or discrete accumulation of *erg11* and/or *cdr1B* mRNAs upon growth in the presence of voriconazole, as possible causes for resistance. Our results suggest that other mechanisms different from target mutation or increased expression are probably influencing voriconazole resistance in these strains. Previously, we have observed that *in vitro* evolution of itraconazole resistance in *A. fumigatus* involves multiple mechanisms of resistance, such as the overexpression of drug efflux pumps (da Silva Ferreira *et al.* 2004). Furthermore, genome sequencing of these mutant strains and SNP comparisons with the parental strains are necessary to understand the mechanisms that are responsible for voriconazole resistance.

In summary, the *A. fumigatus atmA* and *atrA* genes are important for genetic stability and showed strong genetic interaction, but individual mutations do not affect *A. fumigatus* virulence in spite of discrete chromosomal polymorphisms in these mutant strains. However, we have demonstrated that *A. fumigatus* AtmA and AtrA influence the evolution of azole resistance. Additional genomic sequencing studies are necessary to understand the complete array of changes involved in the evolution of voriconazole resistance in these strains.

## Supplementary Material

Supplemental material is available online at www.g3journal.org/lookup/suppl/doi:10.1534/g3.117.300265/-/DC1.
