# Supplementary figures and images for: The Influence of Genetic Stability on Aspergillus fumigatus Virulence and Azole Resistance
Source: G3 (Bethesda). 2017 Nov 17;8(1):265–78. doi: 10.1534/g3.117.300265 (PMC5765354; doi:10.1534/g3.117.300265)

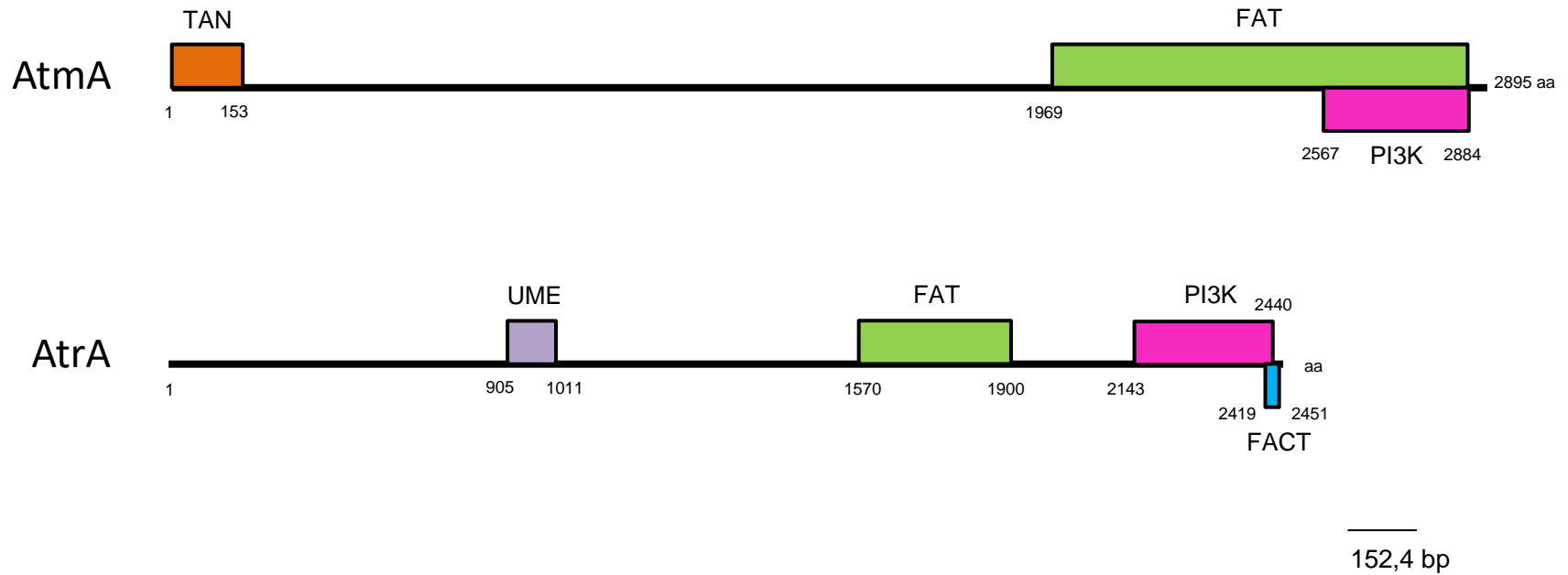

**Supplementary Figure S1-** Putative domains in *A. fumigatus* AtmA and AtrA.

Supplement: Supplementary file 1 [file 265FigureS1.pdf]
