# Supplementary material for: The Influence of Genetic Stability on Aspergillus fumigatus Virulence and Azole Resistance
Source: G3 (Bethesda). 2017 Nov 17;8(1):265–78. doi: 10.1534/g3.117.300265 (PMC5765354; doi:10.1534/g3.117.300265)
Supplement: Supplementary file 2 [file 265FigureS2.pdf]

Afu5g12660 null mutant (Δ*atmA*)

A.

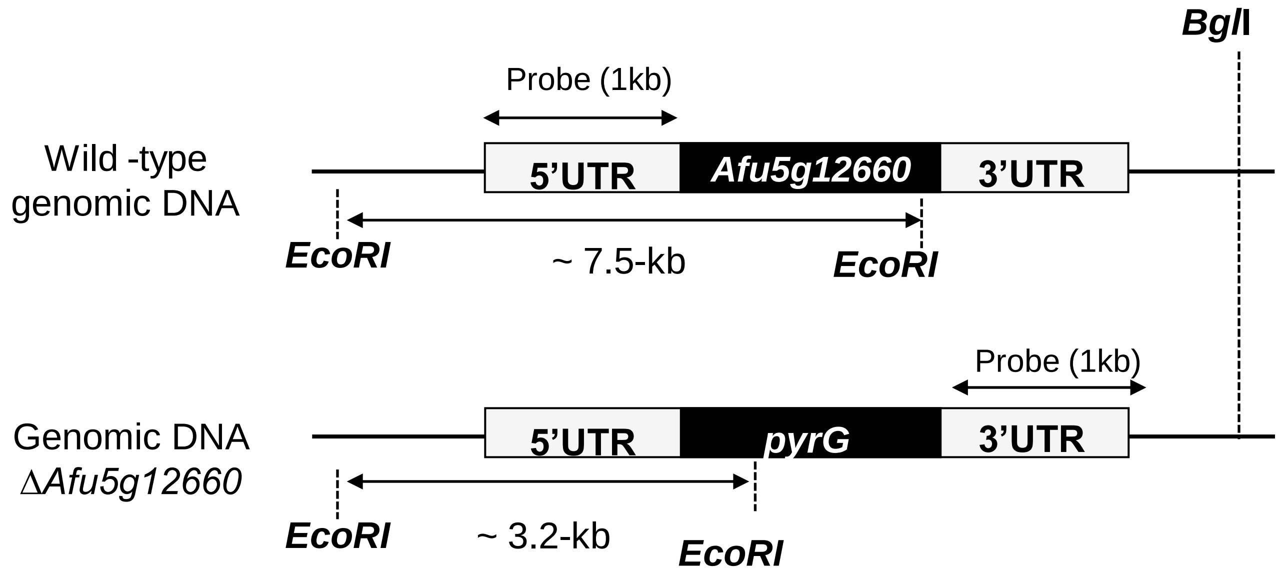

B.

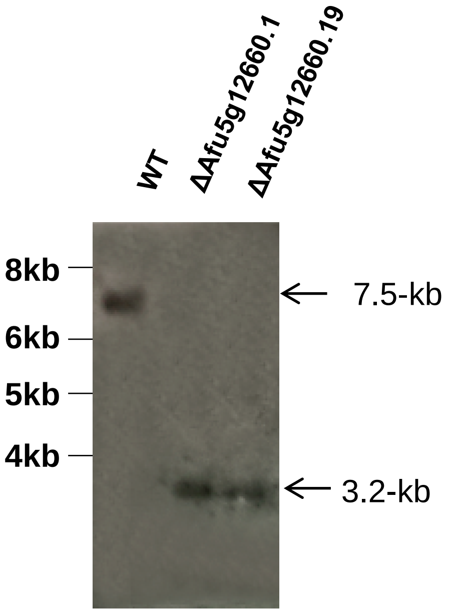

Afu4g04760 null mutant (Δ*atrA*)

A.

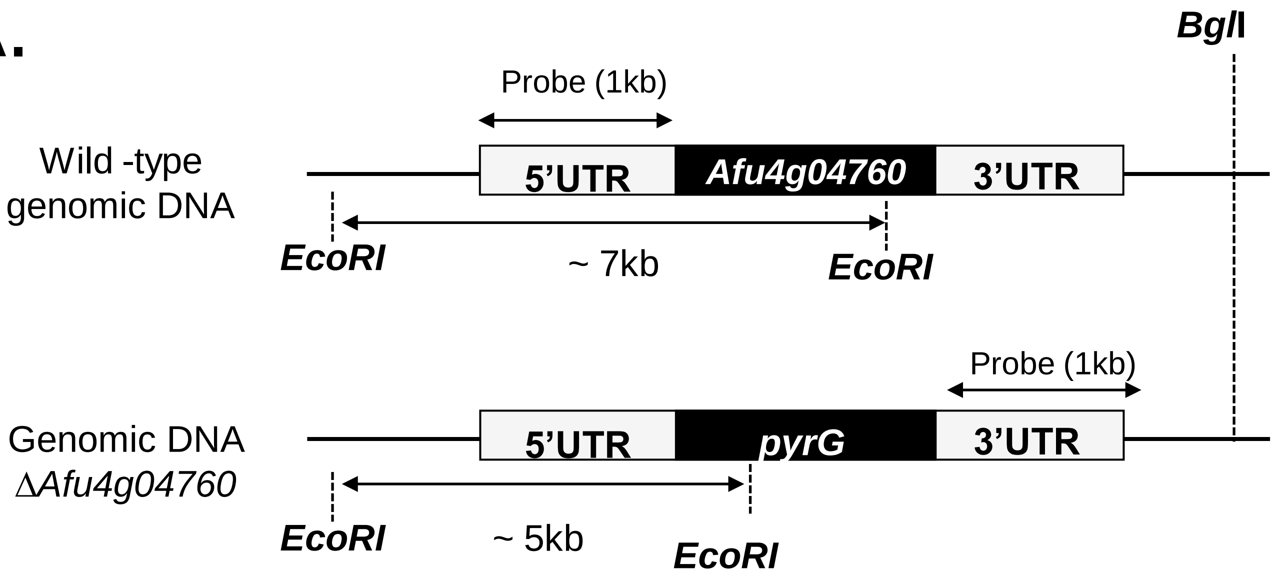

B.

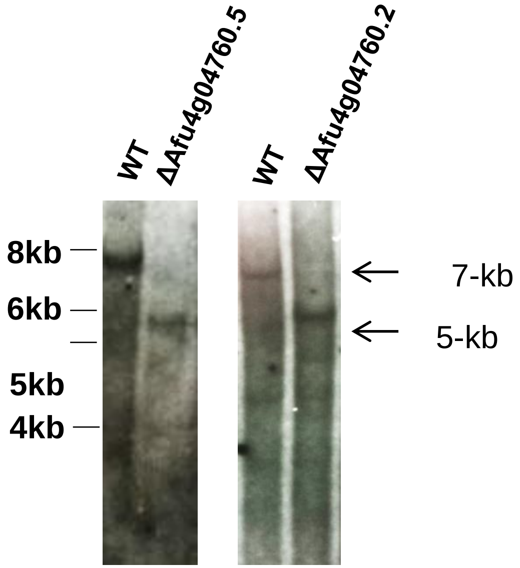

Supplementary Figure S2 - Genomic DNA from the *A. fumigatus* Af293 wild type, Δ*atmA* (Afu5g12660) and Δ*atrA* (Afu4g04760) strains was extracted and digested with the *EcoRI* restriction enzyme in order to confirm the *atmA* and *atrA* deletion strains. A schematic diagram (**A.**) and Southern blot (**B.**) is shown for the wild-type and *ΔatmA* strain when digested with EcoRI. A 1-kb DNA fragment from the *atmA* 5'UTR (untranslated region) was used as a hybridization probe. This probe recognizes a single ~7.5 kb band in the wild-type and a single ~3.2 kb band in the *ΔatmA* strain. The schematic diagram for wild-type and *ΔatrA* strain is shown (**C.**) and the Southern blot (**D.**). After digestion with *EcoR*I, a 1-kb DNA fragment from the *atrA* 5'UTR (untranslated region) was used as a hybridization probe. The probe recognizes a single 7.0 kb band in the wild-type strain and a single 5.0 kb band in the *ΔatrA* strain.
