# Supplementary material for: The Influence of Genetic Stability on Aspergillus fumigatus Virulence and Azole Resistance
Source: G3 (Bethesda). 2017 Nov 17;8(1):265–78. doi: 10.1534/g3.117.300265 (PMC5765354; doi:10.1534/g3.117.300265)
Supplement: Supplementary file 3 [file 265FigureS3.pdf]

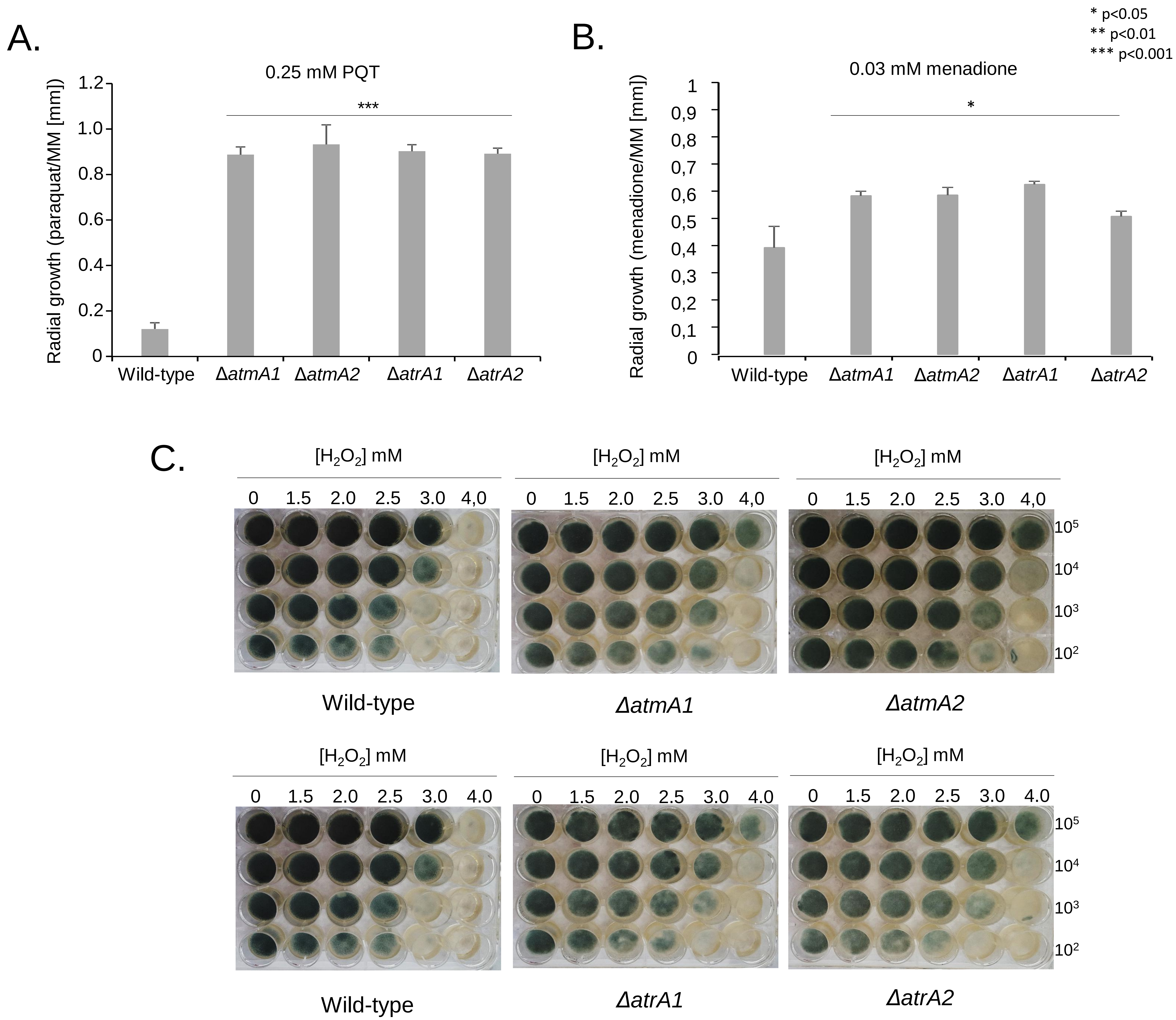
* p<0.05
** p<0.01
*** p<0.001
A.
0.25 mM PQT
Radial growth (paraquat/MM [mm])
1.2
1.0
0.8
0.6
0.4
0.2
0
***
Wild-type
ΔatmA1
ΔatmA2
ΔatrA1
ΔatrA2
B.
0.03 mM menadione
Radial growth (menadione/MM [mm])
1
0,9
0,8
0,7
0,6
0,5
0,4
0,3
0,2
0,1
0
*
Wild-type
ΔatmA1
ΔatmA2
ΔatrA1
ΔatrA2
C.
[H2O2] mM
0 1.5 2.0 2.5 3.0 4,0
10^5
10^4
10^3
10^2
Wild-type
ΔatmA1
ΔatmA2
[H2O2] mM
0 1.5 2.0 2.5 3.0 4.0
Wild-type
ΔatrA1
ΔatrA2

**Supplementary Figure S3 -** The *ΔatmA* and *ΔatrA* mutant strains are more resistant to oxidative stress. Radial growth of the wild-type, ΔatmA and ΔatrA strains was measured after 120h growth in solid MM at 37°C supplemented with (A) 0.25mM Paraquat (PQT) and (B) 0.03mM menadione. The results are the average ± standard deviation of 3 repetitions. Statistical analysis was performed. (C) Ten-fold conidial dilutions ($10^7$ to $10^4$) of the wild-type and the null mutants were grown in YG in the absence or increasing soncentration of $H_2O_2$ concentrations for 48h at 37°C.
