# Supplementary material for: The Influence of Genetic Stability on Aspergillus fumigatus Virulence and Azole Resistance
Source: G3 (Bethesda). 2017 Nov 17;8(1):265–78. doi: 10.1534/g3.117.300265 (PMC5765354; doi:10.1534/g3.117.300265)
Supplement: Supplementary file 4 [file 265FigureS4.pdf]

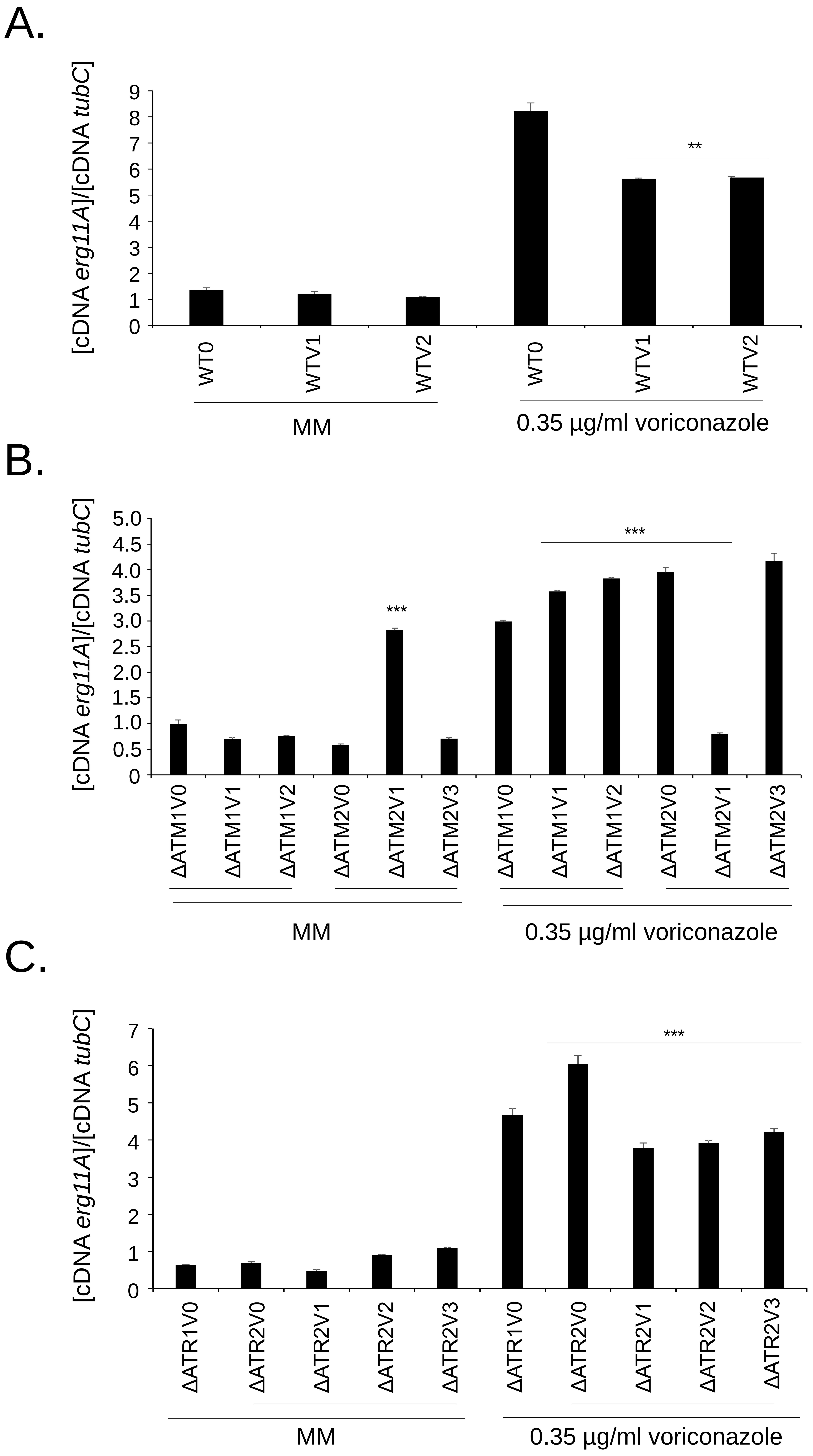
A.
[cDNA erg11A]/[cDNA tubC]
0
1
2
3
4
5
6
7
8
9
**
WT0
WTV1
WTV2
WT0
WTV1
WTV2
MM
0.35 µg/ml voriconazole
B.
[cDNA erg11A]/[cDNA tubC]
0
0.5
1.0
1.5
2.0
2.5
3.0
3.5
4.0
4.5
5.0
***
***
ΔATM1V0
ΔATM1V1
ΔATM1V2
ΔATM2V0
ΔATM2V1
ΔATM2V3
ΔATM1V0
ΔATM1V1
ΔATM1V2
ΔATM2V0
ΔATM2V1
ΔATM2V3
MM
0.35 µg/ml voriconazole
C.
[cDNA erg11A]/[cDNA tubC]
0
1
2
3
4
5
6
7
***
ΔATR1V0
ΔATR2V0
ΔATR2V1
ΔATR2V2
ΔATR2V3
ΔATR1V0
ΔATR2V0
ΔATR2V1
ΔATR2V2
ΔATR2V3
MM
0.35 µg/ml voriconazole

Supplementary Figure S4 – The *erg11A* mRNA accumulation in *A. fumigatus* wild-type and mutant strains. (A-C) *A. fumigatus* strains were grown for 16 hours in MM and transferred to MM+0.35 µg/ml voriconazole for 1 hour. All gene expression was normalized by the amount of β-tubulin (*tubC*). Standard deviations present the average of three independent biological repetitions. Statistical analysis was performed using a one-way ANOVA test by comparing the exposed to respective the non-exposed strains to voriconazole (**, $p < 0.005$ and ***, $p < 0.001$).
