# Supplementary material for: The Influence of Genetic Stability on Aspergillus fumigatus Virulence and Azole Resistance
Source: G3 (Bethesda). 2017 Nov 17;8(1):265–78. doi: 10.1534/g3.117.300265 (PMC5765354; doi:10.1534/g3.117.300265)
Supplement: Supplementary file 6 [file 265FigureS6.pdf]

	Sample Name	Count
	A. nidulans haploid	27237
	A. nidulans diploid	27133
	A. fumigatus WTV1	26931
	A. fumigatus Af293 wild-type	26719
	A. fumigatus ΔATMA2V1	26810
	A. fumigatus ΔATRA2V1	26694

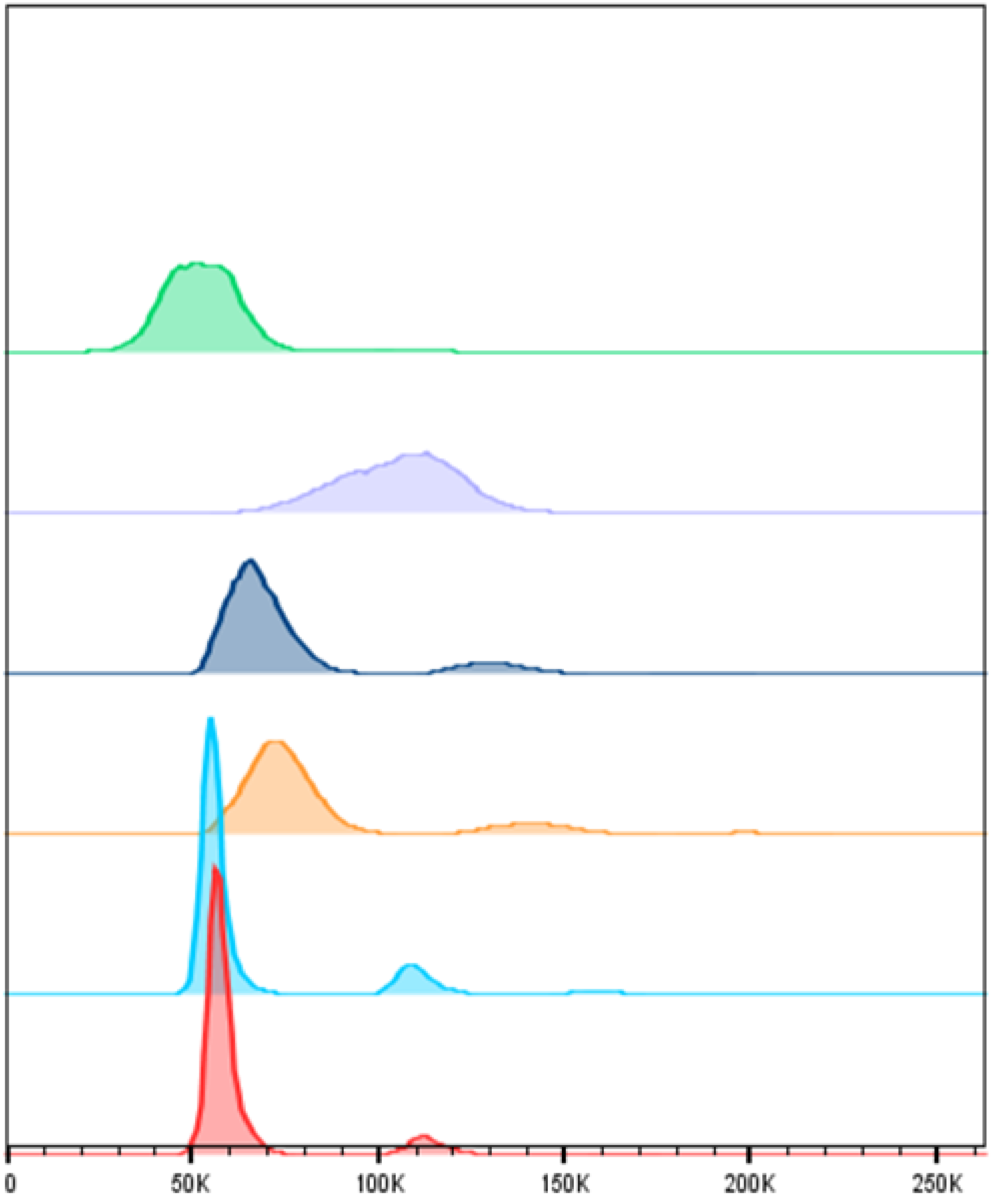

Supplementary Figure S6 - Fluorescence-activated cell sorting (FACS) analysis of *A. fumigatus* and *A. nidulans* DNA content.
