# Supplementary material for: The Influence of Genetic Stability on Aspergillus fumigatus Virulence and Azole Resistance
Source: G3 (Bethesda). 2017 Nov 17;8(1):265–78. doi: 10.1534/g3.117.300265 (PMC5765354; doi:10.1534/g3.117.300265)
Supplement: Supplementary file 7 [file 265TableS1.docx]

**Supplementary Table S1**. Strains and plasmids used in this study

| **STRAINS/PLASMID** | **GENOTYPE** | **REFERENCE** |
| --- | --- | --- |
| ***Plasmid*** |  |  |
| *pRS426* | *ampR lacZ* URA3 | Teepe et al., 2007 |
| ***S. cerevisiae*** |  |  |
| Sc9721 | \| MATa his 3-D200 URA 3-52 leu2D1 lys 2D202 trp 1D63 \|  \| \| --- \| --- \| | FGSC |
| ***A. fumigatus* strains** |  |  |
| Af293 | pyrG | FGSC |
| ΔatmA1 | pyrG; ΔatmA::pyrG | This study |
| ΔatmA2 | pyrG; ΔatmA::pyrG | This study |
| ΔatrA1 | pyrG; ΔatrA::pyrG | This study |
| ΔatrA2 | pyrG; ΔatrA::pyrG | This study |
| Δ*atrA1* *niiA::atmA* | pyrG; ΔatrA::pyrG; atmA::niiA | This study |
