# Supplementary material for: The Influence of Genetic Stability on Aspergillus fumigatus Virulence and Azole Resistance
Source: G3 (Bethesda). 2017 Nov 17;8(1):265–78. doi: 10.1534/g3.117.300265 (PMC5765354; doi:10.1534/g3.117.300265)
Supplement: Supplementary file 8 [file 265TableS2.docx]

**Supplementary Table S2**. Primers used in this study

|  | **name** | **Sequence (5´- 3´)** |
| --- | --- | --- |
| P1 | atmA pRS426 5F | GTAACGCCAGGGTTTTCCCAGTCACGACGGTTTTGATCTCCACGATGAATGC |
| P2 | atmA pyrG 5R | CAGTGCCTCCTCTCAGACAGAATCACGATGGCGCGGCTATCCGTA |
| P3 | atmA pyrG 3F | GAGCATTGTTTGAGGCGAATTCGCACTCAGAAATGTGTAGCAAAG |
| P4 | atmA pyrG 3R | GCGGTTAACAATTTCTCTCTGGAAACAGCTCAGGGATCAGACGGACCAGTC |
| P5 | atrA pRS426 5F | GTAACGCCAGGGTTTTCCCAGTCACGACGGAAATGAAGAAGTGAAACAAGCCG |
| P6 | atrA pyrG 5R | CAGTGCCTCCTCTCAGACAGAATATGTGTAATAGAGAAAGCATCAAAAG |
| P7 | atrA pyrG 3F | GAGCATTGTTTGAGGCGAATTCAAGAGAATTGACCCGTACATCATTG |
| P8 | atrA pyrG 3R | GCGGTTAACAATTTCTCTCTGGAAACAGCGACCAAGAATGTCACCACATCTC |
| P9 | pyrG F | ATTCTGTCTGAGAGGAGGCACTGATGCG |
| P10 | pyrG R | GAATTCGCCTCAAACAATGCTCTTCACC |
| P11 | atmA 5UTR pyrG NiiA R | GCTCGTATTTTTCCCTGCGCTATCCGTATTTCGCACAC |
| P12 | atmA gene R niiA pRS | GCGGATAACA ATTTCACACA GGAAACAGCC TTCTTTAGCT ATGCAATCAA TACC |
| P13 | atmA prom NiiA F: | cgttgagacttcgtcacgATGGCTCAGGTCACTCTTG |
| P14 | cyp51A/erg11A | AAGGAGCAGGAGAACGACAA |
| P15 | cyp51A/erg11A | GCCAGAATCACACCAAGGTT |
| P16 | tubC SYBR F | AGCTGGCGGTAACAAATACG |
| P17 | tubC SYBR R | ACCTGATCCACCAATTCTGC |
